# Profiling Neuroactive Compounds in Organic, Conventional, and Processed Tomatoes

**DOI:** 10.3390/foods14223927

**Published:** 2025-11-17

**Authors:** Ana Kovačič, Mar Garcia-Aloy, Domenico Masuero, Vania Sáez, Anže Brus, Pietro Franceschi, Urška Vrhovšek

**Affiliations:** 1 Metabolomics Unit, Research and Innovation Centre, Fondazione Edmund Mach, 38098 San Michele all’Adige, Italy; 2 Department of Environmental Sciences O2, Jožef Stefan Institute, Jamova 39, 1000 Ljubljana, Slovenia; 3 Digital Agriculture Unit, Research and Innovation Centre, Fondazione Edmund Mach, 38098 San Michele all’Adige, Italy

**Keywords:** tomato, neuroactive chemical profile, neuroprotective compounds, neuro-disrupting compounds, annotation, mass spectrometry

## Abstract

Despite growing evidence linking diet to neurodegenerative diseases, the connection remains unclear. Tomatoes, a widely consumed food, have been proposed as potential sources of neuroactive compounds. Using LC-MS/MS, we profiled organic and conventional “datterini,” plump, and processed tomatoes. Six carotenoids were quantified, with phytoene and lycopene being the most abundant. Multivariate analysis revealed that processing and variety, rather than organic vs. conventional methods, drove dataset variability. Seventy neuroactive compounds were identified, some distinguishing tomato variety, processing, and/or production methods. Processed tomatoes generally showed higher abundance of neuroactive compounds than fresh tomatoes, and “datterini” tomatoes contained more neuroprotective compounds than plump tomatoes. Organic “datterini” did not have higher neuroprotective compound levels than conventional ones. These findings suggest thermal processing may alter the compositional quality of tomatoes, potentially enhancing the levels of certain bioactive constituents, while organic cultivation does not inherently increase the abundance of neuroprotective compounds. Overall, tomatoes represent a complex source of both neuroprotective and neuro-disrupting compounds, warranting further research on their bioaccessibility and physiological relevance.

## 1. Introduction

Neurodegenerative diseases (NDs) pose a significant global health burden, affecting millions of people worldwide [1]. While the exact etiology of these diseases remains complex, emerging evidence suggests a strong correlation between diet and ND risk, particularly with dietary patterns high in fruits, vegetables, and omega-3 fatty acids, and low in processed foods and saturated fats [2]. Adequate intake of specific nutrients or food groups, such as polyphenols, vitamins, and amino acids, has demonstrated notable clinical benefits in both ND patients and preclinical models. Dietary factors may play a crucial role in both disease prevention and progression [2,3].

Tomato (*Solanum lycopersicum* L.) is a widely consumed vegetable globally, celebrated for its versatility, affordability, and impressive nutritional profile. As a key component in the so-called “Mediterranean diet”, consumption of raw tomato and tomato-based products has been associated with various health benefits, including reduced risk of chronic degenerative diseases [4].

The nutritional richness of tomatoes is attributed to a diverse array of bioactive compounds, such as vitamins (C, E, and folate), minerals (potassium), carotenoids (β-carotene and lycopene), polyphenols (quercetin, kaempferol, naringenin, caffeic, ferulic, and chlorogenic acids), and neurotransmitters (serotonin, GABA, and glutamic acid) [5]. These compounds have been associated with various health-promoting effects, including antioxidant, anti-inflammatory, anti-cancer, and neuroprotective activities [6]. It has also been suggested that tomatoes may benefit from the additive and synergistic actions of the various phytochemicals present in their natural food matrix [7].

To date, most research has focused on specific nutrients, such as amino acids, carotenoids, and polyphenols; however, only a limited number of the potentially bioactive plant compounds have been characterized [8], often limiting the scope to a few selected tomato cultivars [6]. Recent studies suggest that vegetables, including tomatoes, may contain neurotoxic compounds, such as pesticides, food additives, and pharmaceutical residues, which can be introduced during production or processing [9,10,11]. The production and processing of tomatoes can also significantly influence the presence and concentration of bioactive compounds. Factors like environmental conditions, cultivar, and processing techniques can impact the overall neuroactive profile of the final product [6]. The available literature suggests that organically produced tomatoes and smaller-size tomato varieties may offer higher food quality, though the evidence is mixed. Similarly, the quality of processed versus fresh tomatoes is debated, with studies often focusing on compounds with beneficial nutritional effects [4,12]. Existing research tends to examine neuroprotective and neurotoxic compounds in isolation, overlooking potential interactions between these substances [13]. Non-targeted analysis, particularly mass spectrometry (MS), provides accurate mass values of peaks obtained through high-accuracy mass measurements and, if available, MS/MS fragmentation patterns, which offer abundant annotation for each peak. This approach has been valuable for understanding the chemical profiles in food; however, much remains unclear, and the identities of most ion peaks detected by MS are still unknown [8].

To address these knowledge gaps, this study aimed to investigate the comprehensive neuroactive profile of organically and conventionally grown tomatoes, as well as different types and processed tomato products. By employing advanced analytical techniques, including targeted (HPLC-DAD-MS) and non-targeted (UHPLC-QToF-MS) approaches, we identified a wide range of neuroactive compounds, encompassing both beneficial (neuroprotective) and potentially harmful (neuro-disrupting) compounds. This research provides valuable information on the neuroactive compounds present in tomatoes, contributing to a better understanding of the chemical composition people may be exposed to through tomato consumption. Furthermore, the results of this study will lay the base for our future research aimed at understanding the fate of neuroactive compounds once digested and their potential impact on the gut microbiome.

## 2. Material and Methods

### 2.1. Chemicals and Standard Preparation

A total of 52 target neuroactive compounds and 4 isotopically labelled compounds and rosmarinic acid were used for spiking and quality control experiments (Table S1), including neurotransmitters (*n* = 6), pharmaceuticals (*n* = 2), pesticides (*n* = 7), mycotoxins (*n* = 1), industrial chemicals (*n* = 8), hormones (*n* = 2), polyphenols (*n* = 8), plant alkaloids (*n* = 3), antibiotics (*n* = 3), carotenoids (*n* = 10) and steroid (*n* = 1). The selection of compounds was based on their known or potential neuroactivity, both protective and disruptive, the diversity of their chemical structures and physicochemical properties (including polarity and molecular size), their primary biological action, their potential occurrence in tomatoes, and their availability in the laboratory at the time of analysis. All analytical standards and internal standards (carbamazepine-d_10_, 4-hydroxyphenyl-^13^C_6_, serotonin-d_4_, ^13^C_12_-bisphenol S, rosmarinic acid) were obtained from Merck (Steinheim, Germany) or TransMIT, Dr Ehrenstorfer GmbH (Darmstadt, Germany) and CanSyn Chem. Corp. (Toronto, ON, Canada), and were of high purity (>90%). Individual stock standard solutions (~1.0 mg/mL) were prepared by dissolving each compound in methanol or combination of methanol/water and carotenoids in ethyl acetate. Working standards (1.0 and 10 µg/mL) were prepared by appropriate serial dilutions of the stock solutions. Similarly, an internal standard solution (5.0 µg/mL) was prepared from a solution (~1.0 mg/mL) of isotopically labelled compounds and rosmarinic acid (~1.54 mg/mL) was used as external standard for carotenoid analysis. Standard solutions were stored in amber glass vials at −20 °C or −80 °C (carotenoids). All analytical grade solvents and ammonium formate (NH_4_CHO_2_, >99.0%) were obtained from Merck and formic acid (HCOOH, >99%) from Chebios (Rome, Italy). Ultrapure water (18.0 MΩ cm at 25 °C) was prepared using the Milli-Q water purification system. Samples were filtered using Verex Filter Vial Kit, RC 0.2 μm (Phenomenex, Torrance, CA, USA), and 0.22 μm PTFE membrane filters.

### 2.2. Study Design

The experimental design included four different groups of tomato: organically and conventionally produced “datterini” (DO and D, respectively), fresh plump tomatoes (PF, all San Marzano variety), and processed tomato (PS, tomato sauce made from plump tomatoes). The tomato products were purchased from a local supermarket in Trento, Italy, from six different sources (different producers or supermarkets) for each group, resulting in 6 experimental food replicates per group. The collected tomato samples were cut, freeze-dried, and stored at room temperature in boxes (protected from light) containing silica beads. The water content of all tomato samples was 92 ± 2%. Detailed information is provided in Table S2.

### 2.3. UHPLC-MS-DAD Analysis of Carotenoids

#### 2.3.1. Sample Preparation

Due to structural properties, carotenoids required different sample preparation and instrumentation. The extraction of carotenoids was modified based on the procedure described by Multari et al. [14,15], where the method was also fully validated. Sample preparation was performed under dim light. Freeze-dried tomato (200 ± 1 mg) was extracted into 5 mL of a methanol/acetone/hexane mixture (25/25/50%, *v*/*v*/*v*), vortexed, and mixed on an orbital shaker for 10 min. Extraction was performed in an ultrasonic bath at 10 °C and 59 kHz for 10 min. The mixtures were then centrifuged (10 min, 1800× *g*, 4 °C), and the organic layers were transferred to 50 mL clean flasks. This step was repeated three times. The organic extracts were combined and dried under nitrogen at ≤35 °C. The dry extracts were saponified overnight at room temperature in a shaking incubator, using 4 mL of a 15% KOH solution in methanol (*w*/*v*). After saponification, 3.5 mL of NaCl solution (9%, *w*/*v*) and 4 mL of hexane:diethyl ether (3:1, *v*/*v*) were added to the samples. The mixtures were placed on an orbital shaker for 10 min at room temperature, vortexed, and centrifuged (5 min, 1800× *g*, 4 °C). This step was repeated twice. The organic layers were combined and washed three times with 5 mL of water, then dried under reduced pressure at ≤35 °C and reconstituted in 0.25 mL of a MTBE/EtOH mixture (1/2, *v*/*v*) containing rosmarinic acid at 20 µg/mL. Samples were filtered through 0.22 μm PTFE membranes and analyzed. Quality control (QC) samples were prepared by pooling aliquots of each sample (100 ± 1 mg) and prepared identically to individual samples. The analysis of separate tomato parts was performed in the same way, i.e., using the same amount of tomato peel and core, as the total amount of tomato sample (200 ± 1 mg).

#### 2.3.2. Method Optimization

Due to poor ionization, the carotenoid analysis was performed on an UPLC–MS system equipped with a diode array detector (DAD) and a quadrupole mass spectrometer (UPLC-PDA-qDA, Waters, Milford, MA, USA). The entire system was controlled by MassLynx version 4.2 (Waters, Milford, MA, USA). The method, adopted from Dumont et al. [16], was modified for the quantification of ten carotenoids and lupeol. Chromatographic separation was achieved on a BEH C18 polymer column (1.7 µm, 2.1 × 100 mm), maintained at 55 °C. The mobile phases consisted of acetonitrile/water (1:1, *v*/*v*) (eluent A) and isopropanol (eluent B), both containing 0.1% (*v*/*v*) formic acid and buffered with 10 mM ammonium formate. The carotenoids were eluted with the following gradient: initial conditions were 0.25 mL/min, 65% A; down to 45.7% A in 4 min; then down to 36.1% A in 0.5 min; isocratic elution for 3.5 min; down to 0% A in 6 min, held for 1.5 min. A cleaning method was performed before returning to the initial conditions: three consecutive injections of 10 µL of a blank solution (MTBE:EtOH, 1:2, *v*/*v*) using the following elution gradient at 0.25 mL/min: two times for 0.6 min with 100% B, and third blank injection for 0.6 min with 100% B then back to the initial conditions (65% A) in 3 min. The total run time (sample run plus cleaning method) was 20.3 min. The sampler operated at 15 °C. The DAD acquisition ranged from 270 to 600 nm in 1.2 nm steps. The mass spectrometer was equipped with an ESI source at 150 °C in positive ion mode with a 15 V cone voltage. Mass acquisitions were performed in full scan mode (100–1200 *m*/*z*) and SIR mode. For carotenoid quantification in tomato fruits, injections of 1 µL were performed with concentrated extract and diluted extract (1/10 or 1/20 in MTBE/EtOH 1:2, *v*/*v*). Data acquisition was performed in a random injection order and a quality control sample was injected every 10 samples to evaluate potential carry-over and instrument signal stability.

Linear external calibrations, prepared with 6–7 points and injected in duplicate, were used to quantify carotenoids using the DAD λmax calibration curve at working ranges of 1.56–100 µg/g dry weight (dw) or 3.83–250 µg/g dw tomato. The obtained LOQ (limit of quantification) were 1.56 or 3.83 µg/g dw (0.078/0.192 mg/kg fresh weight (fw)) tomato, and the extraction recovery ranged from 64% to 150%. The method and instrumental repeatability were <10.1% and 15.5%, respectively. The quality control samples confirmed no analytical variability, with the variation in signal being less than 17%. Detailed information is provided in Table S3.

### 2.4. UHPLC-QTOF-MS Non-Target Analysis

#### 2.4.1. Sample Preparation

Lyophilized tomato samples (m = 0.25 g) were weighed, and 40 µL of internal standard mixture (5 µg/mL) was added along with methanol (V = 0.3 mL) to achieve a final internal standard concentration of 0.3 ppm in the vial. The mixture was vortexed and shaken on an orbital shaker for 15 min. A QC sample was prepared by pooling each sample (m = 0.1 g) and spiking it with the final internal standard mixture concentration of 0.3 ppm and a mixture of non-labelled neuroactive compounds to final concentrations of 1.6 µg/g dry weight (dw). The list of the standards used for QC is presented in Table S1. Different extraction solvents, ultrasound conditions, solid-phase extraction methods, and reconstitution solvents were tested to optimize sample preparation and acquisition. The optimized method was then applied to acquire the untargeted dataset as follows: The homogenate was sonicated for 15 min at room temperature and centrifuged for 10 min. The supernatant was transferred and evaporated under a nitrogen flow. Finally, the residue was reconstituted with 0.3 mL ultrapure water/methanol (90/10, *v*/*v*) and filtered through a 0.2 μm RC filter.

#### 2.4.2. Method Optimization

Instrumental analysis was performed using ultra-performance liquid chromatography quadrupole-time-of-flight mass spectrometry (UPLC-qToF-MS) (Synapt XS, Waters Corporation, Milford, MA, USA). An ACQUITY Premier HSS T3 Column (1.8 µm, 2.1 × 100 mm) was used at 40 °C and eluted with a binary mobile phase of ultrapure water containing 0.1% formic acid (A) and methanol containing 0.1% formic acid (B) at a flow rate of 0.3 mL/min following the gradient program: 0 min, 95% A; 0–1 min, 95% A; 1–3 min, 80% A; 4–12 min, 20% A; 12–16 min, 0% B; 16–18 min, 0% B; 18.0–18.1 min, 95% A; and 18.1–20.0 min, 95% A. The injection volume of the sample extract was 2 μL. After each injection, the needle was rinsed with 500 μL of weak wash solution (water/methanol, 90:10). Samples were kept at 10 °C during the analysis. The data acquisition modes were MS^E^ continuum and centroid DDA acquisition. In the DDA method, both full scan and MS/MS spectra of the three most intense ions from each full scan were acquired. All experiments were conducted in both ESI (+) and ESI (−) ionization modes. The source and desolvation temperatures were 120 °C and 450 °C, respectively, with core and desolvation gas flow rates of 50 and 800 L/h. The capillary voltage was 3 kV for ESI (+) and 2.5 kV for ESI (−) experiments. The cone voltage was set at 40 V. Collision energy was set to 6 eV (trap) for low-energy scans, with 20–40 eV ramps employed for ESI (−) and ESI (+) in high-energy scans. The data acquisition range was 50–1500 Da. Leucine-enkephalin was continuously infused for lock mass correction (200 pg/L, 10 µL/min, 0.2 s scan time, 15 s interval) as the reference (*m*/*z* 556.2766 ESI (+) and 554.2620 ESI (−)).

#### 2.4.3. Data Acquisition Quality

The non-targeted method was developed and optimized using QC samples. Method validation parameters, including matrix effect, extraction efficiency, and both method and instrumental repeatability, were assessed for 41 neuroactive compounds, comprising 25 neuro-disrupting and 16 neuroprotective compounds, spiked into QC samples at two concentrations (0.1 µg/g and 1.6 µg/g dw). A matrix-matched calibration curve, containing both the internal standard mixture and target compounds, was prepared in the QC samples at concentrations of 0.03, 0.07, 0.13, 0.27, 0.53, 0.80, 1.60, and 2.67 µg/g dw. Solvent blanks, consisting of pure solvent injections and procedural blanks (methanol), were used, and QC samples were injected at the beginning of the sequence for instrumental calibration. Multiple injections of the QC samples were also made throughout the analytical run to evaluate the stability and performance of the analytical system, focusing on retention time drift, mass accuracy, signal intensities, carryover phenomena, and potential analytical variations. To evaluate the quality of the acquired data, raw files were examined manually using MassLynx Mass Spectrometry Software (Waters) and an *in-house* developed script in R (R version 4.4.2) to extract the ion chromatogram of target compounds during acquisition. The recoveries of the tested compounds ranged from 67% to 157% at the higher concentration (1.6 µg/g dw) and from 41% to 130% at the lower concentration (0.1 µg/g dw). Although some values exceeded 100%, these were mainly observed for a few compounds at the lower concentration level, likely due to matrix effects close to the detection limit. This information was taken into account during data pre-processing, where analytical drift was corrected, and during the annotation of mass features. Method and instrumental repeatability, assessed by the relative standard deviation of triplicates, were found to be <20% and <15%, respectively. For most compounds, with a few exceptions, the R^2^ of the linear calibration curve was >0.9, and the matrix effect was ± 60%. No carryover was observed based on multiple injections of solvent and procedural blanks. The principal component analysis (PCA) analysis grouped solvents and procedure blanks separately from the samples and also clustered the QC samples together (Figure S1). Detailed information is provided in Table S4.

#### 2.4.4. Data Processing and Compound Annotation

The LC-MS dataset is publicly available (accession number: REQ20250205208439) on MetaboLights repository (https://www.ebi.ac.uk/metabolights/editor/study/REQ20250205208439, accessed on 13 November 2025) [17]. Raw data were converted into mzML format using ProteoWizard software. Data processing, performed using Progenesis QI, resulted in a two-dimensional dataset, where each mass feature was characterized by its specific retention time (RT) and mass-to-charge ratio (*m*/*z*), with signal intensities quantified by calculating the peak areas for each feature. Double injections with two different mass spectrometry acquisition methods enabled the collection of high-quality full scan data during the first injection with the MS^E^ mode, dedicated to further statistical analysis, while the second injection with the DDA modality allowed for the automatic collection of a high number of qualitative MS/MS spectra. All *in-house* developed R codes (R version 4.4.1) for data processing and statistical analysis, along with the generated mass feature table, are available on GitHub (https://github.com/ana-kovacic/NeuroTOm, accessed on 13 November 2025). Analytical drifts were removed for each features applying a linear regression normalisation on the log transformed intensities (Figure S2). After drift removal, missing values were imputed by replacing them with a random value between zero and one-tenth of the minimum value within the corresponding feature (i.e., minimum area of individual mass features between all samples). Additional feature filtering steps were applied to exclude noisy and non-relevant mass features from the subsequent analysis. In-depth details can be found in the section “Data Acquisition Quality and Data processing” in the Supporting Information.

Compound annotation was performed focusing on identifying neuroactive compounds. Initially, compounds were identified by comparing *m*/*z* values, retention times, and MS/MS spectra to those of available analytical standards. The confidence level for these identifications corresponds to Level 1 (L1). To expand the annotation beyond available standards, we employed a suspect screening approach, utilizing an extended in-house database compiled from authoritative online sources (e.g., NORMAN Substance Database, Humanneurotox) and peer-reviewed literature. Compounds were classified as potentially neuroactive based on their documented or predicted effects on the nervous system, whether beneficial (neuroprotective) or adverse (neuro-disrupting). It is important to note that this classification was based solely on the reported associations in the literature or database entries, and not on a direct assessment of their neurophysiological impact within this study. Mass features were annotated by matching the *m*/*z* and experimental MS/MS spectral data with MS/MS data from spectral libraries such as MassBank and GNPS, and by manually evaluating matching chemical structures for individual peaks. The confidence level for these annotations corresponds to Level 2 (“putatively annotated compound”) and Level 3 (“putatively characterized compound”) when experimental MS/MS data and chemical knowledge were available, but no MS/MS database was available for comparison, as defined by the Metabolomics Standards Initiative [18].

### 2.5. Statistical Analysis

Statistical analyses were performed at the level of the individual features. Pairwise comparisons were conducted on both the target data (quantified carotenoids) and non-target data (annotated compounds) across the following groups: D vs. DO (organically produced “datterini” vs. conventional “datterini”), D vs. PF (conventional “datterini” vs. plump tomato), and PS vs. PF (processed plump tomatoes vs. fresh plump tomatoes). These comparisons allowed for the identification of characteristic mass features for organic production, tomato variety, and processing, as well as non-discriminating compounds present across all tomato groups. A compound was considered discriminant for a specific condition on the base of the following criteria: absolute value of Cohen’s d > 1 (effect size) and *p*-value < 0.05 (independent *t*-test). Using both parameters allowed to highlight compounds that were both statistically significant and showed a contrast in abundance between the compared groups. Specifically, discriminant compounds were marked as “+” for higher abundance, “−” for lower abundance, and “=” for non-discriminant compounds.

Additionally, PCA was applied to the non-target data to explore associations between different tomato sample groups and identify patterns that could distinguish between the tested conditions.

## 3. Results and Discussion

### 3.1. Characterization of the Neuroactive Compound Profile in Four Types of Tomatoes

#### 3.1.1. Carotenoids

**Figure 1:**
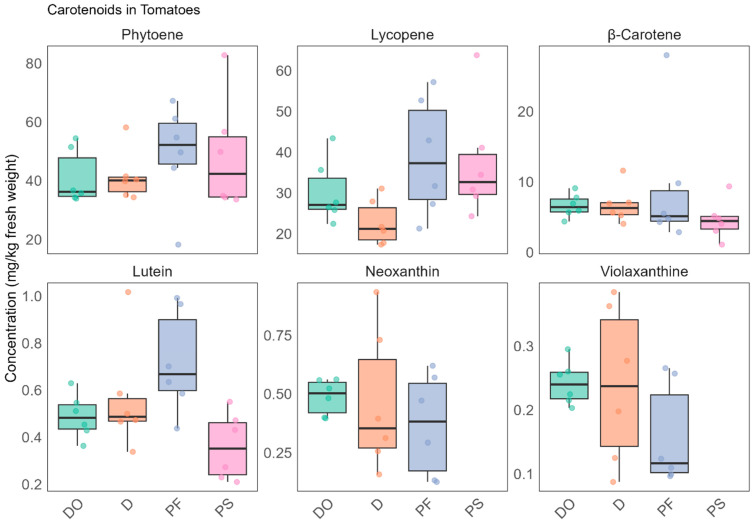
Carotenoid’s concentration in different tomato types (*n* = 6/group, each colored dot represents an experimental tomato food replicate): DO—organically produced “datterini”, D—conventionally produced “datterini”, PF—fresh plump tomatoes, PS—processed tomatoes.

Six out of the ten carotenoids were quantified and the results are displayed in Figure 1. Phytoene (18.2–82.7 mg/kg fresh weight (fw)) and lycopene (17.3–63.8 mg/kg fw) were the most abundant carotenoids, followed by β-carotene (1.1–28.0 mg/kg fw). Lutein was present at lower concentrations (0.2–1.0 mg/kg fw), while neoxanthin and violaxanthin were detected at even lower levels (<LOQ–0.93 mg/kg fw) and were not detected in the processed tomato samples (Table S5). Overall, the determined concentrations are generally consistent with previously reported values in tomatoes [19,20]. Lycopene and phytoene were found at the highest levels, in agreement with the literature (lycopene: 10–180 mg/kg fw; phytoene: up to ~30 mg/kg fw), while β-carotene and lutein were present at lower levels (β-carotene: 1–12 mg/kg fw; lutein: ≤1 mg/kg fw), and neoxanthin and violaxanthin were detected at trace amounts. Slight differences, such as the slightly higher phytoene concentrations observed in our study, may be explained by the presence of multiple isomers of phytoene or by differences in tomato varieties, growing conditions, and sample handling.

Pairwise comparisons were conducted on the detected compounds to identify discriminant compounds among different tomato groups. A compound was considered significantly different between two groups if it met both of the following criteria: absolute value of Cohen’s d > 1 and *p*-value < 0.05. The results of these comparisons (Table S6) showed no significant differences in carotenoid distribution between organically and conventionally produced “datterini” tomatoes (DO vs. D), nor between conventional “datterini” and plump tomatoes (D vs. PF). The only exception was lutein, which was a discriminant compound between processed tomatoes and fresh plump tomatoes (PS vs. PF).

**Figure 2:**
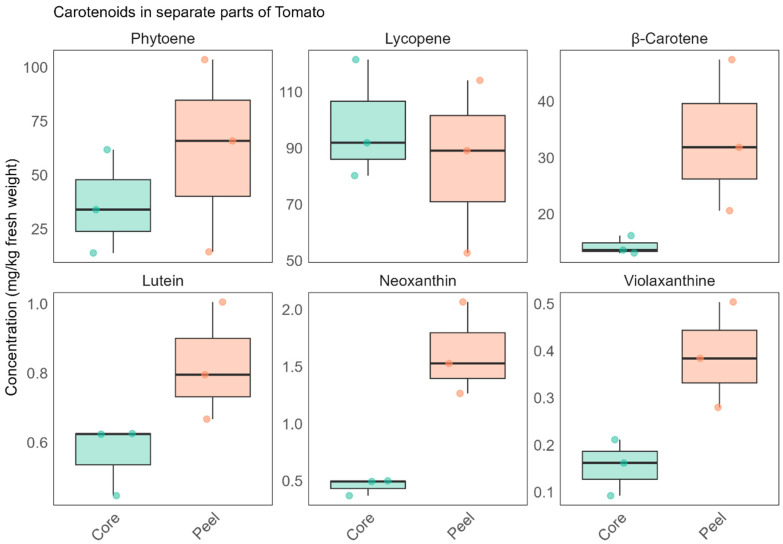
Distribution of carotenoids in separate parts of tomatoes (*n* = 3/group, each colored dot represents an experimental tomato food replicate): Core represents the concentration of carotenoids in the tomato pulp, while Peel indicates their concentration in the skin.

Since the peel is typically removed during processing, we hypothesise that the observed reduction in the levels of lutein, violaxanthin, and neoxanthin in processed tomatoes compared to fresh tomatoes may be partly attributed to the loss of these carotenoids during peeling. However, as processed tomatoes are generally free of peel and the process also involves thermal treatment, this effect should be regarded as a possible contributing factor rather than a definitive cause. To further explore this hypothesis, a complementary analysis was conducted to investigate whether the absence of peel contributes to the lower carotenoid content. In this analysis, the peel and pulp (core) of fresh plump tomatoes (*n* = 3, m = 0.1 g of freeze-dried peel or core) were separated and analysed individually. The results revealed that carotenoids, particularly neoxanthin, violaxanthin, lutein, and β-carotene, were present at higher concentrations in the peel compared to the pulp (Figure 2). However, although carotenoid concentrations are higher in the peel, it is important to note that the peel accounts for only about 5–10% of the total tomato weight, depending on fruit size (lower for larger tomatoes, higher for smaller ones) [21]. For instance, a 100 g serving of raw, normal-sized tomato would provide approximately 0.05 ± 0.008 mg of lutein from the pulp, compared to only 0.008 ± 0.001 mg from the peel. Thus, the difference in carotenoid content between processed and fresh tomatoes may be attributed more to the processing itself rather than solely to the removal of the peel.

#### 3.1.2. Identification of Neuroactive Compounds

The PCA of the entire mass feature dataset indicates that the main source of variability in the data is associated with tomato processing, since PS samples were separated along the first component from the other analysed samples; whereas the second and orthogonal source of variability was associated with the tomato variety since PC2 separated “datterini” tomato (mixing together both conventional and organic ones) from plump tomato (Figure S3).

Beyond the PCA, the filtered mass feature list, which was matched against an *in-house* generated database of potential neuroactive compounds in tomatoes, resulted in the annotation of 70 compounds at varying confidence levels (Table 1): 30 were identified at level 1, 33 at level 2, and 7 at level 3. In the absence of an analytical standard, the possible isomers were presented as (I, II), based on the same precursor ion, MS/MS, and close retention times. The derivative of acetaminophen was putatively identified as such because its MS/MS pattern matched to that obtained from the injection of the analytical standard of acetaminophen, even if the retention time differed. The detailed information regarding the identification of compounds is provided in the Supplementary Materials, including their fragmentation spectra (“MSMS_Neuroactive profile in tomatoes.pdf”). These MS/MS spectra were matched in the following ways: First, the spectra from tomato samples were compared to those of analytical standards, with common fragments highlighted in red. Second, they were matched with spectra from online MS/MS libraries, with shared fragments highlighted in blue. Finally, spectra with no match in the library are also provided. Additionally, the Supplementary Materials includes boxplots showing the normalized intensities (on a log_10_ scale, Table S7) of each identified compound across all tested sample groups (DO, D, PF, and PS), which can be found in the file “DistributionNeuroactiveCompoundsTomatoes.pdf”.

Among the identified compounds there were both neuroprotective (62) and neuro-disrupting (8) species, which were subsequently categorized into nine classes based on their chemical structure and/or use. In particular, neuroprotective compounds were classified into the following classes: amino acids and derivatives (*n* = 10), fatty acids (*n* = 5), neurotransmitters (*n* = 6), nucleotides and nucleosides (*n* = 5), phenolic compounds/polyphenols and derivatives (*n* = 27), plant alkaloids and secondary metabolites (*n* = 7), vitamins, and coenzymes (*n* = 2). The neuro-disrupting, compounds were instead categorized as industrial chemicals (*n* = 4), and food additives (*n* = 4).

**Figure 3:**
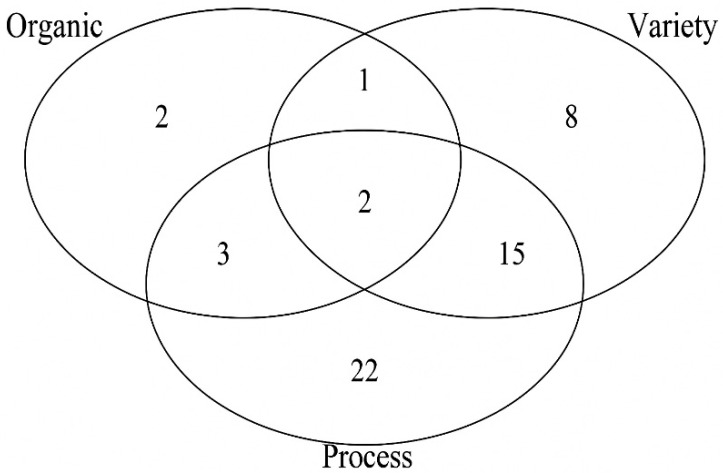
Venn diagram showing the discriminant indicators for one or more tested comparisons: Organic (organic “datterini” vs. conventional), Variety (conventional “datterini” vs. plump), Process (processed tomatoes vs. fresh plump tomatoes).

The identified compounds were categorized as indicators of organic production, variety, and/or processing using the same statistical approach as for the carotenoids, as detailed in Section 4. Eight compounds were discriminant for organic tomatoes, 26 for tomato variety, and 42 for tomato processing. Notably, while some of these “discriminant” compounds were shared across multiple comparisons, others were unique. Specifically, 2 compounds were exclusive as discriminant for organic tomatoes, 8 for tomato variety (i.e., distinguishing “datterini” from plum tomatoes), and 22 for tomato processing. This distribution is illustrated in the Venn diagram in Figure 3.

**Figure 4:**
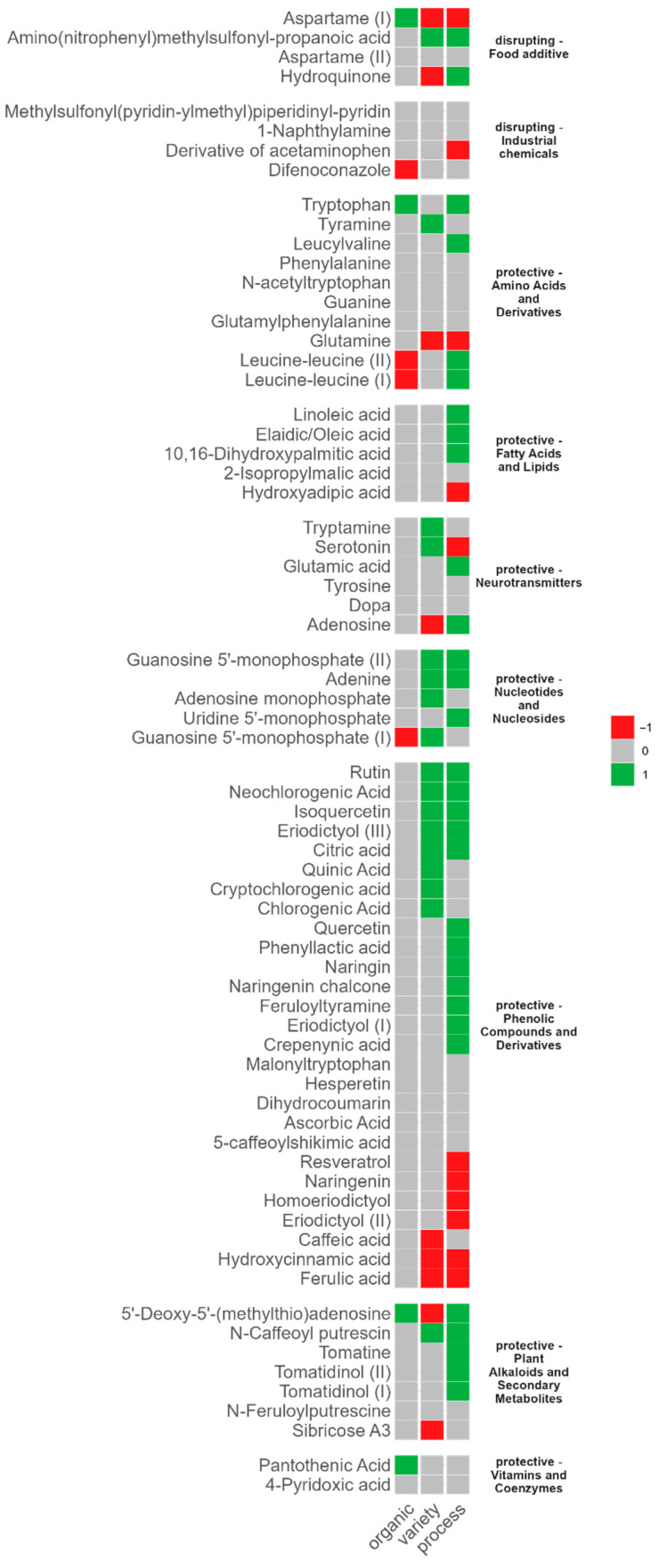
Heatmap illustrating the distribution of identified neuro-disrupting and neuroprotective compounds, grouped by class, based on statistical significance and abundance contrast between compared groups. Discriminant compounds for organically produced tomatoes (Organic: DO vs. D), tomato variety (Variety: D vs. PF), or processed tomatoes (Process: PS vs. PF) were assigned a “+” (red tile) for higher abundance or “−” (green tile) for lower abundance. Non-discriminant compounds for any tested condition were marked with “=” (grey tile).

To identify potential common trends in the abundance patterns within the compound classes the results of the individual comparisons were summarised as follows: (i) a compound with higher abundance in organic “datterini” compared to conventional “datterini” was marked with a “+”, and lower abundance was highlighted with a “−” (organic: DO vs. D); (ii) a compound with higher abundance in “datterini” compared to plump tomatoes was also marked with “+”, while lower abundance was assigned “−” (variety: D vs. PF); (iii) a compound with higher abundance in processed plump tomatoes compared to fresh plump tomatoes was marked with “+”, while lower abundance was assigned “−” (process: PS vs. PF). Those cases in which the compound was not discriminant for any of the performed comparisons were marked as “=”. In Figure 4, the patterns in “+”, “−”, and “=” are represented using a three color heatmap.

Among the neuro-disrupting compounds, food additives showed a diverse presence pattern. For example, aspartame (I), an artificial additive not naturally found in tomatoes, was detected as a discriminant for all tested conditions. Its presence may reflect potential contamination during cultivation or handling rather than a natural component of the fruit. Interestingly, aspartame exhibited higher levels in organic “datterini” compared to conventional, lower levels in “datterini” compared to plump tomatoes, and slightly lower levels in processed tomatoes, which may be partly due to degradation or loss during processing. Among industrial chemicals, the derivative of acetaminophen was identified as a unique discriminant compound for processed tomatoes due to its lower presence compared to fresh plump tomatoes. Pesticides are known to be one of the main pollutants that threaten tomato crops. Interestingly, difenoconazole, one of the most frequently used fungicides and extensively regulated [22], resulted in being a discriminant compound for organic tomatoes due to its lower presence in organically produced tomatoes. For the other two annotated industrial chemicals (i.e., 1-naphthylamine and methylsulfonyl(pyridin-ylmethyl)piperidinyl-pyridin), no specific distribution among tomato groups was observed. So far, the literature has primarily focused on distinguishing organic vs. conventional and fresh versus processed tomatoes based on bioactive compounds with positive effects. However, harmful chemicals in tomatoes, as reported by Ramos et al. (2021) [11], were also detected at trace levels in our study, warranting further investigation.

Regarding neuroprotective compounds, the majority were found to be associated with processing, with higher presence in processed tomatoes compared to fresh tomatoes (PS vs. PF). Discriminatory compounds were also showing higher abundance in conventional “datterini” compared to plum tomatoes (D vs. PF). Some compounds also showed higher abundance in organic “datterini” compared to conventional ones (i.e., tryptophan, 5′-Deoxy-5′-(methylthio)adenosine, and pantothenic acid) while others showed lower abundance (i.e., both leucine-leucine isomers, and one of the two guanosine 5′-monophosphate isomers) in organic “datterini” compared to conventional “datterini” (DO vs. D).

The class of amino acids and derivatives resulted in a very diverse pattern of discriminant compounds, while, fatty acids, except for hydroxyadipic acid, exhibited a higher abundance in processed tomatoes.

The discriminant neurotransmitters indicated differences in variety or processing; for example, tryptamine and serotonin were higher in “datterini” tomatoes compared to plum tomatoes, while adenosine was lower. Additionally, glutamic acid and adenosine were higher and serotonin was lower in processed tomatoes.

Interestingly, nucleotides discriminated based on both variety and processing, primarily due to their higher presence in “datterini” and processed tomatoes, respectively. Similarly, polyphenols mainly discriminated based on variety and processing, with no compounds distinguishing organic tomatoes from conventional ones. Plant alkaloids also showed a strong tendency to discriminate processed tomatoes, mainly due to their higher presence compared to fresh plum tomatoes. Three alkaloids also discriminated conventional ‘datterini’ from plum tomatoes. Sibricose and 5′-Deoxy-5′-(methylthio)adenosine were present at lower levels in ‘datterini,’ while caffeoyl putrescine was more abundant in ‘datterini’ compared to plum tomatoes. Of the two identified vitamins/coenzymes, only pantothenic acid was observed as a discriminant compound for organic tomatoes, showing higher presence in organically produced “datterini.”

In contrast to most studies that report higher carotenoid levels in larger and fresh tomatoes [6] our results did not show significant differences in carotenoid distribution. Although previous studies have reported that organically produced tomatoes contain statistically higher (*p* < 0.05) levels of phenolic compounds compared to conventionally produced ones [23,24], this difference was not statistically significant in our study. Specifically, the contact of most neuroprotective compounds did not significantly differ between conventional and organically produced “datterini” tomatoes (Figure 4, organic–grey). Thus, the trend toward higher phenolic content in organic tomatoes was not observed, whereas difenoconazole, a neuro-disrupting compound, was detected in lower concentrations in the organic samples. This discrepancy with previous studies may be due to differences in cultivar, growing conditions, post-harvest handling, or sample size, which can all influence phytochemical content.

As far as the variety is concerned, tomato varieties of smaller size, such as cherry tomatoes and, in our case, “datterini” tomatoes, are known to be characterized by higher levels of specific potentially bioactive compounds, including total polyphenols and carotenoids, while they are reported to contain lower amounts of amino acids and lipids compared to larger, heavier varieties, such as plum tomatoes [6]. In general agreement with these reports, in our study discriminant neuroprotective compounds showed higher levels in “datterini” tomatoes compared to plum tomatoes, particularly within the nucleotide and polyphenol classes. Some compounds, however, showed an opposite trend, as presented in Figure 4 (e.g., glutamine, sibricose, caffeic acid, ferulic acid), whereas carotenoids did not significantly discriminate smaller from larger varieties.

The effect of processing, particularly thermal treatment, on phytochemicals is a controversial topic in the literature [4]. Some studies suggest a significant loss of hydrophilic antioxidants due to heat exposure, while others report increased bioavailability for certain compounds, such as carotenoids, fatty acids, and polyphenols [25,26]. Our results suggest that processed tomatoes were not of lower “quality” in terms of their neuroactive compound profile, as most discriminant neuroprotective compounds exhibited comparable or even higher relative abundances in processed tomatoes, particularly annotated fatty acids, polyphenols, and plant alkaloids, based on statistical comparisons of relative abundances. A possible explanation is the release of compounds from the matrix during processing, as suggested for polyphenols [27] and for fatty acids, where the observed increase during thermal processing has been attributed to the degradation of the tomato cell wall and the inactivation of enzymes responsible for fatty acid degradation, such as those acting on linoleic acid [25]. Moreover, no higher levels of neurotoxic compounds were observed in processed tomatoes, contrary to our initial hypothesis.

Overall, the presence of important neuroprotective compounds, such as carotenoids, amino acids, fatty acids, nucleotides, polyphenols, alkaloids, and vitamins, was confirmed, each exhibiting diverse biological activities, including anti-inflammatory, anti-amyloidogenic, anti-cholinesterase, hypolipidemic, and antioxidant effects [28]. Although these compounds were identified in tomatoes, their bioavailability and metabolic fate after digestion remain to be further investigated [29], representing an important direction for future research. Neurotransmitters, which are key neuroprotective compounds, were also identified and found to discriminate among the tested tomato groups. For instance, tryptamine and serotonin were more abundant in “datterini” tomatoes, while glutamic acid and adenosine showed higher levels in processed tomatoes. Other neurotransmitters, such as DOPA, an important dopamine precursor, were present without relevant differences between tomato groups. Some clinical trials have reported the bioavailability of dietary neurotransmitters, such as glutamate, and their potential health effects [30]. However, while neurotransmitters are not expected to cross the blood–brain barrier directly, they can be transferred indirectly. Importantly, our study confirmed the presence of neuro-disrupting compounds alongside neuroprotective ones. The fate of these compounds during digestion and fecal fermentation remains largely unknown. For context, some compounds detected in our study, such as difenoconazole and aspartame, have [31,32,33] been reported to be bioaccessible in the colon or to interact with neural pathways. Therefore, the presented results should be interpreted with caution, and further studies are needed to better understand human chemical exposure through food.

## 4. Conclusions

The optimized analytical methods and workflows developed in this study allowed a detailed characterization of the neuroactive compound profiles in different tomato-based products, considering both neuroprotective and neuro-disrupting compounds. The results indicate that the primary factors affecting the compound profiles are the processing methods and the tomato varieties, rather than the type of production (organic vs. conventional). The analysis identified 70 neuroactive compounds and quantified 6 carotenoids, some of which were found to be discriminant for the different factors taken into consideration in our study.

Overall, organically produced “datterini” tomatoes do not appear to have a higher concentration of the annotated neuroprotective compounds compared to conventionally produced ones. However, “datterini” tomatoes, when compared to plum tomatoes, appear to have a more favourable neuroactive profile. Interestingly, processed tomatoes generally show a higher abundance of neuroactive compounds compared to fresh tomatoes, based on the compounds annotated in this study. These findings suggest that processed tomatoes may retain valuable bioactive compounds, challenging the assumption that organic tomatoes are inherently more neurohealth-promoting than conventional ones or that processing necessarily reduces nutritional value.

The identified profiles suggest that tomato products, as a food source, have a rich neuroprotective compound profile, although the presence of neuro-disrupting compounds was also confirmed and should not be overlooked. Special attention should be given to the fact that the detection of neuro-disrupting compounds may be missed due to their lower concentration which could be below the instrumental limit of detection. These compounds could potentially have significant effects at much lower concentrations compared to neuroprotective compounds. Therefore, the presented results should be considered carefully, and further efforts are needed to deepen our understanding of human chemical exposure through food.

We acknowledge the limitations of our sampling design, particularly the relatively small sample size and the fact that all samples were collected from a single city (Trento), which together limit the generalizability of our findings. Importantly, the primary aim of this study was to characterize the neuroactive profiles across organically and conventionally grown tomatoes, as well as different types and processed tomato products, by establishing a robust screening platform. This analysis provides a comprehensive overview of the compounds to which consumers may be exposed through tomato consumption, highlighting candidates for future studies on their fate during digestion and potential effects on the gut microbiome. While bioavailability was beyond the scope of this study, these results lay the groundwork for ongoing investigations into the transformation and absorption of key neuroactive compounds within the human gastrointestinal tract and fecal fermentation. The findings offer valuable insights into the neuroactive compounds present in tomatoes, enhancing our understanding of the compounds we are exposed to through the consumption of various tomato products. This research provides evidence that can help clarify the relationship between diet and the development of neurodegenerative diseases. However, it also underscores the need for further research to thoroughly investigate human exposure to neuroactive compounds via food consumption. Specifically, it will be crucial to examine the fate of these compounds within the human body to better understand their potential links to neuropsychiatric effects and neurotoxicity. Future studies should integrate multi-omics data to provide a more comprehensive understanding, which will be the focus of our upcoming research efforts.

## Figures and Tables

**Table 1:** Summary of identified compounds, including their neuroactivity, classification, measured precursor mass (*m*/*z*) for ion adduct, their retention time (RT) [min], confidence level (L), and results of the individual comparisons (statistical analysis). The symbol + or − indicate a higher or lower presence, respectively, and are used to identify discriminant compounds in organically produced tomatoes (Organic: DO vs. D), tomato variety (Variety: D vs. PF), and processing method (Process: PS vs. PF). A “=” sign denotes non-discriminant compounds.

Neuro-Activity	Class	Compound	*m*/*z*	RT [min]	Ion Adduct	Level	Organic	Variety	Process
disrupting	Food additive	Amino(nitrophenyl)methylsulfonyl-propanoic acid	289.052	3.25	[M + H]^+^	L3	=	+	+
		Aspartame (I)	295.1154	6.2	[M + H]^+^	L1	+	−	−
		Aspartame (II)	295.1297	5.43	[M + H]^+^	L2	=	=	=
		Hydroquinone	109.0296	3.97	[M − H]^−^	L2	=	−	+
	Industrial chemicals	1-Naphthylamine	144.0812	4.56	[M + H]^+^	L2	=	=	=
		Derivative of acetaminophen	152.0709	1.58	[M + H]^+^	L3	=	=	−
		Difenoconazole	406.0727	13.41	[M + H]^+^	L1	−	=	=
		Methylsulfonyl(pyridin-ylmethyl)piperidinyl-pyridin	332.1436	12.07	[M + H]^+^	L3	=	=	=
protective	Amino Acids and Derivatives	Glutamine	147.0773	0.87	[M + H]^+^	L2	=	−	−
		Glutamylphenylalanine	295.1299	3.70	[M + H]^+^	L1	=	=	=
		Guanine	152.0709	1.58	[M + H]^+^	L1	=	=	=
		Leucine-leucine (I)	245.1846	5.96	[M + H]^+^	L1	−	=	+
		Leucine-leucine (II)	245.1867	5.91	[M + H]^+^	L1	−	=	+
		Leucylvaline	231.1711	4.96	[M + H]^+^	L1	=	=	+
		N-acetyltryptophan	245.0922	6.88	[M − H]^−^	L2	=	=	=
		phenylalanine	166.0861	3.4	[M − H]^−^	L2	=	=	=
		Tryptophan	205.0976	4.51	[M − H]^−^	L2	+	=	+
		Tyramine	138.0902	1.95	[M − H]^−^	L2	=	+	=
	Fatty Acids and Lipids	10,16-Dihydroxypalmitic acid	287.2223	12.04	[M − H]^−^	L2	=	=	+
		2-Isopropylmalic acid	175.0609	5.42	[M − H]^−^	L2	=	=	=
		Elaidic/Oleic acid	281.2471	16.57	[M − H]^−^	L3	=	=	+
		Hydroxyadipic acid	161.0453	3.6	[M − H]^−^	L1	=	=	−
		Linoleic acid	279.2332	16.1	[M − H]^−^	L2	=	=	+
	Neurotransmitters	Adenosine	268.1041	2.59	[M + H]^+^	L1	=	−	+
		Dopa	198.0744	2.01	[M + H]^+^	L1	=	=	=
		Glutamic acid	148.0614	0.87	[M + H]^+^	L1	=	=	+
		Serotonin	177.1027	2.74	[M + H]^+^	L1	=	+	−
		Tryptamine	161.1080	4.60	[M + H]^+^	L2	=	+	=
		Tyrosine	182.0811	1.91	[M + H]^+^	L1	=	=	=
	Nucleotides and Nucleosides	Adenine	136.0623	1.16	[M + H]^+^	L2	=	+	+
		Adenosine monophosphate	346.0540	1.32	[M + H]^+^	L2	=	+	=
		Guanosine 5′-monophosphate (I)	362.0507	1.23	[M − H]^−^	L2	−	+	=
		Guanosine 5′-monophosphate (II)	362.0507	1.69	[M − H]^−^	L2	=	+	+
		Uridine 5′-monophosphate	323.0289	1.14	[M − H]^−^	L2	=	=	+
	Phenolic Compound/Polyphenols and Derivatives	5-caffeoylshikimic acid	335.0783	6.36	[M + H]^+^	L2	=	=	=
		Ascorbic Acid	191.0191	1.30	[M + H]^+^	L2	=	=	=
		Caffeic acid	179.0352	5.74	[M − H]^−^	L1	=	−	=
		Chlorogenic Acid	353.0878	5.27	[M − H]^−^	L1	=	+	=
		Citric acid	191.0189	1.31	[M − H]^−^	L1	=	+	+
		Crepenynic acid	277.2194	15.71	[M − H]^−^	L2	=	=	+
		Cryptochlorogenic acid	353.0886	5.34	[M − H]^−^	L1	=	+	=
		Dihydrocoumarin	147.0441	3.41	[M − H]^−^	L2	=	=	=
		Eriodictyol (I)	287.0562	8.68	[M − H]^−^	L2	=	=	+
		Eriodictyol (II)	287.0564	9.04	[M − H]^−^	L2	=	=	−
		Eriodictyol (III)	287.0550	6.99	[M − H]^−^	L3	=	+	+
		Ferulic acid	193.0500	5.14	[M − H]^−^	L1	=	−	−
		Feruloyltyramine	341.1390	8.72	[M − H]^−^	L1	=	=	+
		Hesperetin	301.0725	9.66	[M − H]^−^	L1	=	=	=
		Homoeriodictyol	301.0726	9.97	[M − H]^−^	L1	=	=	−
		Hydroxycinnamic acid	163.0399	2.02	[M − H]^−^	L1	=	−	−
		Isoquercetin	463.1240	8.06	[M − H]^−^	L2	=	+	+
		Malonyltryptophan	289.0828	6.90	[M − H]^−^	L1	=	=	=
		Naringenin	271.0607	9.58	[M − H]^−^	L1	=	=	−
		Naringenin chalcone	271.0639	9.51	[M − H]^−^	L2	=	=	+
		Naringin	581.1870	7.67	[M − H]^−^	L2	=	=	+
		Neochlorogenic Acid	353.0874	4.22	[M − H]^−^	L1	=	+	+
		Phenyllactic acid	165.0551	6.96	[M − H]^−^	L1	=	=	+
		Quercetin	301.0363	9.58	[M − H]^−^	L2	=	=	+
		Quinic Acid	191.0553	1.0	[M − H]^−^	L2	=	+	=
		Resveratrol	227.0714	7.0	[M − H]^−^	L3	=	=	−
		Rutin	609.1462	7.99	[M − H]^−^	L1	=	+	+
	Plant Alkaloids and Secondary Metabolites	5′-Deoxy-5′-(methylthio)adenosine	298.0973	4.73	[M + H]^+^	L3	+	−	+
		N-Caffeoyl putrescin	251.1392	4.02	[M + H]^+^	L3	=	+	+
		N-Feruloylputrescine	265.1548	5.10	[M + H]^+^	L2	=	=	=
		Sibricose A3	461.1302	3.73	[M + H]^+^	L2	=	−	=
		Tomatidinol (I)	414.3376	8.69	[M + H]^+^	L1	=	=	+
		Tomatidinol (II)	414.3376	8.86	[M + H]^+^	L2	=	=	+
		Tomatine	1034.5546	10.14	[M − H]^−^	L2	=	=	+
	Vitamins and Coenzymes	4-Pyridoxic acid	182.0455	2.72	[M − H]^−^	L1	=	=	=
		Pantothenic Acid	220.1185	4.23	[M + H]^+^	L1	+	=	=

## Data Availability

The data is shared through the researcher’s repositories in accordance with the FAIR principles (as open as possible). Raw data is available in the publicly accessible MetaboLights repository (accession number: REQ20250205208439) at https://www.ebi.ac.uk/metabolights/editor/study/REQ20250205208439 (accessed on 13 November 2025), and R codes are available on GitHub at https://github.com/ana-kovacic/NeuroTOm.git (accessed on 13 November 2025).

## Supplementary Materials

The following supporting information can be downloaded at: https://www.mdpi.com/article/10.3390/foods14223927/s1, References [17,34] are cited in the Supplementary Materials.
