# Supplementary material for: Profiling Neuroactive Compounds in Organic, Conventional, and Processed Tomatoes
Source: Foods. 2025 Nov 17;14(22):3927. doi: 10.3390/foods14223927 (PMC12651413; doi:10.3390/foods14223927)
Supplement: Supplementary file 1 [file foods-14-03927-s001.zip › supplementary-foods-3957470.pdf]

Supplementary information

Profiling Neuroactive Compounds in Organic, Conventional, and Processed Tomatoes

Materials and methods

General information of compounds

Table SI 1 General information about the standards and internal standards used for method validation.

Compound name	Formula	Neuroactive activity	Group
Amoxicillin	C16H19N3O5S	disrupting	antibiotics
Azithromycin	C38H72N2O12	disrupting	antibiotics
Erythromycin	C37H67NO13	disrupting	antibiotics
Estrone (E1)	C18H22O2	disrupting	hormones
β-Estradiol (E2)	C18H24O2	disrupting	hormones
Bisphenol S	C12H10O4S	disrupting	industrial chemicals
Bisphenol F	C13H12O2	disrupting	industrial chemicals
Methylparaben	C8H8O3	disrupting	industrial chemicals
Bisphenol A	C15H16O2	disrupting	industrial chemicals
Dimethyl phthalate (DMP)	C10H10O4	disrupting	industrial chemicals
2,4-di hydroxy benzophenone (DHBP (I))	C13H10O3	disrupting	industrial chemicals
4,4-di hydroxy benzophenone (DHBP (II))	C13H10O3	disrupting	industrial chemicals
Diisodecyl phthalate (DiDP)	C28H46O4	disrupting	industrial chemicals
Alternariol monomethyl ether (AME)	C15H12O5	disrupting	mycotoxins
Methamidophos	C2H8NO2PS	disrupting	pesticides
Imidacloprid	C9H10ClN5O2	disrupting	pesticides
Acetamiprid	C10H11ClN4	disrupting	pesticides
Thiacloprid	C10H9ClN4S	disrupting	pesticides
Dimetamorph (I)	C21H22ClNO4	disrupting	pesticides
Dimetamorph (II)	C21H22ClNO4	disrupting	pesticides
Difenoconazole	C19H17Cl2N3O3	disrupting	pesticides
Carbamazepine	C15H12N2O	disrupting	pharmaceuticals
Diclofenac	C14H11Cl2NO2	disrupting	pharmaceuticals
Ibuprofen	C13H18O2	disrupting	pharmaceuticals
Solanine	C45H73NO15	disrupting	plant alkaloid
Tomatine	C50H83NO21	disrupting	plant alkaloid
Caffeine	C8H10N4O2	disrupting	plant alkaloid
Violaxanthin	C40H56O4	protective	carotenoid
Neoxanthin	C40H56O4	protective	carotenoid
Antheraxanthin	C40H56O3	protective	carotenoid
Lutein epoxide	C40H56O3	protective	carotenoid
Zeaxanthin	C40H56O2	protective	carotenoid
Lutein	C40H56O2	protective	carotenoid
Canthaxanthin	C40H52O2	protective	carotenoid
Lycopene	C40H56	protective	carotenoid
α-carotene and β-carotene	C40H56	protective	carotenoid
Phytoene trans isomers	C40H64	protective	carotenoid
Dopamine	C8H11NO2	protective	neurotransmitters
Tyramine	C8H11NO	protective	neurotransmitters
Serotonin	C10H12N2O	protective	neurotransmitters
Tryptamine	C10H12N2	protective	neurotransmitters
Kynurenic acid	C10H7NO3	protective	neurotransmitters
Melatonin	C13H16N2O2	protective	neurotransmitters
Naringin	C27H32O14	protective	phenolic compounds
Quercetin	C15H10O7	protective	phenolic compounds
Rutin	C27H30O16	protective	phenolic compounds
Genistein	C15H10O5	protective	phenolic compounds
Kaempferol	C15H10O6	protective	phenolic compounds
Isoliquiritigenin	C15H12O4	protective	phenolic compounds
Chlorogenic acid	C16H18O9	protective	phenolic compounds
Resveratrol	C14H12O3	protective	phenolic compounds
Lupeol	C30H50O	protective	steroid
d4-Seratonin	C10H9D4N2OCl		internal standard
d10-Carbamazepine	C15D10H2N2O		internal standard
13C6-Resveratrol	13C6C8H12O3		internal standard
13C12-BPS	13C12H10O4S		internal standard

Study design

The tomato products were purchased from a local supermarket in Trento, Italy, from six different sources (different producers or supermarkets) for each group, resulting in 6 experimental food replicates per group (Table SI 2). The water content of all tomato samples was 92 ± 2%.

Table SI 2 Details of tomato samples collected from various supermarkets in Trento City, specifying the brand, sample name, and water content (%) for each type.

Type of tomato	Sample Name	% Water	Information (Shop Name, City, Brand)
Datterini conventional (D)	D1-1	90.3	Conad, Le Motte, Trento city
	D1-2	90.8	
	D2-1	91.1	Conad, Pomodoro Cocktail Datterino (Prodotto Italiano), Trento City
	D2-2	91.3	
	D3-1	90.9	Eurortaggia, Bauere, Trento city
	D3-2	90.1	
	D4-1	90.4	Coop, Fior fiore, Trento City
	D4-2	90.4	
	D5-1	91.5	Mercato, Fior Fiore, Trento city
	D5-2	92.1	
	D6-1	86.7	Interpoli, Fogliati.com, Trento city
	D6-2	91.2	
Datterini organic (DO)	DO1-1	91.8	Lidle, Le Terre di Ecor (Datterini Bio Organic), Trento City
	DO1-2	91.9	
	DO2-1	91.2	Mercato Orvea, Datterini ViaVerde Bio Prima, Trento City
	DO2-2	92.6	
	DO3-1	89.5	Natura, Econatura (Daterrino Bio Italy Sicilia), Trento City
	DO3-2	90.3	
	DO4-1	93.5	InterPoli, Datterini ViaVerde Bio Prima, Trento City
	DO4-2	94.9	
	DO5-1	91.0	Natura 2, Econatura (Daterrino Bio Italy Sicilia), Trento City
	DO5-2	96.2	
	DO6-1	93.8	Lidle 2, Le Terre di Ecor (Datterini Bio Organic), Trento City
	DO6-2	93.6	
Pelatti- San Marzano-fresh (PF)	PF1-1	95.1	IneterPoli, Pellati pomdoro oblungc rosso, pomodoro delio chef, Trento City
	PF1-2	95.3	
	PF2-1	95.4	EuroSpin, Pellati San Marzanno, Refill, Trento City
	PF2-2	95.3	
	PF3-1	94.2	Liddle, Pellati San Marzanno, Refill, Trento City
	PF3-2	94.2	
	PF4-1	94.4	Mercato Orvea, Pellati San Marzano Cilia, Trento City
	PF4-2	94.3	
	PF5-1	95.9	InterPoli, Pellati San Marzano Cilia, Trento City
	PF5-2	95.5	
	PF6-1	93.1	Mercato, Pelatti San Marzanno, Refill, Trento City
	PF6-2	91.7	
	PF7-1	93.1	EuroSpin 2, Pellati San Marzanno, Refill, Trento City
	PF7-2	93.6	
Pelatti- San Marzano-sause (PS)	PS1-1	92.7	InterPoli, Mutti (Pomodori Pelati San Marzano), Trento City
	PS1-2	92.7	
	PS2-1	92.6	Coop, CASAR (Pomodori Pelati San Marzano), Trento City

	PS2-2	92.4	
	PS3-1	92.2	InterPoli, PrimiaP, (Pomodori Pelati San Marzano), Trento City
	PS3-2	92.1	
	PS4-1	91.9	Conad, Pomodoro Italian (Pomodori Pelati San Marzano), Trento City
	PS4-2	92.0	
	PS5-1	92.3	Coop, Pomodoro Italian (Pomodori Pelati San Marzano), Trento City
	PS5-2	92.0	
	PS6-1	90.5	Conad, Regione che vai Campania (Pomodoro San Marzano), Trento City
	PS6-2	90.5	

UHPLC-MS-DAD Analysis of carotenoids

Method optimization

The method parameters for the developed UHPLC-DAD-MS method used to quantify 10 carotenoids and lupeol are provided in Table SI-2. The optimized method achieved sufficient validation parameters, including limit of quantification, linearity, extraction efficiency, and instrument and method repeatability, as expressed by the RSD between three replicate samples.

Table SI 3. Method performance of a robust analytical method for determining carotenoids in tomato fruit sample, including LOQ, linearity, extraction efficiency, method and instrumental repeatability.

Compound Name	RT (min)	DAD λmax	SIM M+H	Working range		LOQ		Linearity			Extraction efficiency	Instrumental repeatability (15 µg/g dw)	Method repeatability (15 µg/g dw)
				µg/ml in the vial extract	µg/g dw	µg/g dw	mg/kg fw	k	n	R2	RE %	RSD %	
Violaxanthin	2.64	442	601.3	1.25-80	1.56-100	1.56	0.125	0.04	-0.01	0.99	128 ± 31	1.4	0.4
Neoxanthin	2.71	439	601.5	1.25-80	1.56-100	1.56	0.125	0.02	0.01	1.00	110± 21	7.9	5.7
Antheraxanthin	3.34	450	585.5	1.25-80	1.56-100	1.56	0.125	0.11	-0.02	0.96	64 ± 23	2.9	15.5
Lutein epoxide	3.48	440	585.5	1.25-80	1.56-100	1.56	0.125	0.19	-0.03	1.00	150 ± 11	2.1	2.8
Zeaxanthin	4.13	455	569.5	1.25-80	1.56-100	1.56	0.125	0.04	0.00	1.00	106 ± 8	10.1	2.1
Lutein	4.34	448	569.5	1.25-80	1.56-100	1.56	0.125	0.15	-0.02	1.00	98 ± 15	7.9	0.5
Canthaxanthin	5.16	482	565.5	1.25-80	1.56-100	1.56	0.125	0.05	0.05	0.99	86 ± 13	2.1	4.4
Lupeol	6.74	-	427.5	1.25-80	1.56-100	1.56	0.125	0.004	0.02	0.98	130 ± 29	3.2	1.9
Lycopene	10.93	472	537.5	3.06-200	3.83-250	3.83	0.306	0.01	0.01	0.98	93 ± 5	8.8	3.3
α-carotene and β-carotene	11.36	454	537.5	3.06-200	3.83-250	3.83	0.306	0.01	0.06	0.99	87	6.6	2.6
Phytoene trans isomers	11.5	286	545.5	3.06-200	3.83-250	3.83	0.306	0.01	0.00	0.99	92 ± 13	3.0	5.9
Rosmarinic acid	0.81	330	361.5										

UHPLC-QTOF-MS Non-target analysis

Method optimization

UHPLC-QToF-MS was developed and optimized based on 41 selected neuroactive compounds. The method considered extraction efficiency, matrix effect, and method and instrumental repeatability

parameters at two concentration levels: low (0.1 µg/g dw, LL) and high (1.6 µg/g dw, HL). The obtained method and validation parameters are provided in Table SI-3.

Table SI 4. Method performance of analytical method for non-target analysis of neuroactive compounds in tomato fruit at low (0.1 µg/g dw, LL) and high (1.6 µg/g dw, HL) concentration level.

Compound	RT (min)	Formula	Matrix Effect HL	Matrix Effect LL	Recovery LL	Recovery HL	Method Repeatability LL	Method Repeatability HL	Instrumental Repeatability LL	Monoisotopic mass	[M+H]+	$[M-H]^-$
Bisphenol S	7.28	C12H10O4S	-17.74		108.69	98.11	2.55	6.17	3.76	250.0300	251.0373	249.0227
Bisphenol F	7.82	C13H12O2			107.82		8.33	4.48		200.0837	201.0910	199.0765
Methylparaben	7.87	C8H8O3	-24.58		106.46	92.76	1.88	6.41	5.09	152.0473	153.0546	151.0401
Bisphenol A	8.07	C15H16O2			90.74	128.33	7.36	10.45	13.16	228.1150	229.1223	227.1078
2,4-di hydroxy benzophenone (DHBP (I))	8.20	C13H10O3	-12.66		115.00	99.82	1.67	7.33	1.66	214.0630	215.0703	213.0557
4,4-di hydroxy benzophenone (DHBP (II))	10.90	C13H10O3	-11.73		128.63		3.62	3.96		214.0630	215.0703	213.0557
Alternariol monomethyl ether (AME)	12.41	C15H12O5			121.59	95.51	2.51	1.79	2.84	272.0685	273.0757	271.0612
Estrone (E1)	12.58	C18H22O2	-13.52	-100.98	125.21	91.20	50.38	18.34	20.47	270.1620	271.1693	269.1547
Ibuprofen	12.86	C13H18O2		-143.27	93.33	139.41	15.24	17.66	14.40	206.1307	207.1380	205.1234
β-Estradiol (E2)	13.44	C18H24O2	-57.05	0.16	134.07	55.45	10.68	11.65	4.42	272.1776	273.1849	271.1704
Chlorogenic acid	5.29	C16H18O9		-1.86	104.13	98.98	1.85	0.73	4.23	354.0951	355.1024	353.0878
Kynurenic acid	5.30	C10H7NO3	-85.45	-110.97	93.26	80.93	2.85	4.69	10.05	189.0426	190.0499	188.0353
Melatonin	7.53	C13H16N2O2	-21.90	1.42	109.21	77.64	3.71	11.09	7.28	232.1212	233.1285	231.1139
Naringin	7.83	C27H32O14		0.20	106.93	141.18	2.22	3.62	13.12	580.1792	581.1865	579.1719
Quercetin	7.87	C15H10O7	-186.11	0.72	125.72	96.32	2.77	5.03	12.87	302.0427	303.0499	301.0354
Rutin	7.90	C27H30O16		-0.91	104.50	100.12	1.56	2.96	8.60	610.1534	611.1607	609.1461
Resveratrol	8.08	C14H12O3	-9.92		133.99	125.39	3.71	11.23	13.16	228.0786	229.0859	227.0714
Genistein	9.93	C15H10O5	-17.47		111.55	93.70	1.19	3.97	1.79	270.0528	271.0601	269.0455
Kaempferol	10.34	C15H10O6	-14.52		112.78	40.85	1.29	5.72	13.89	286.0477	287.0550	285.0405
Isoliquiritigenin	10.52	C15H12O4	-10.63		126.85	81.18	3.44	7.34	18.44	256.0736	257.0808	255.0663
13C6-Resveratrol	8.08	13C6C8H12O3								234.0988	235.1060	
13C12-BPS	7.28	13C12H10O4S								262.0702	263.0775	
Methamidophos	2.62	C2H8NO2PS	-35.11	-146.14	108.12	80.94	4.02	17.82	20.26	141.0013	142.0086	139.9941
Caffeine	5.70	C8H10N4O2	-37.21	-161.72	112.90	92.09	4.67	16.45	10.87	194.0804	195.0877	193.0731
Amoxicillin	6.12	C16H19N3O5S	-36.13	0.96	112.75	153.64	5.69	25.98	7.80	365.1045	366.1118	364.0973
Imidacloprid	6.42	C9H10ClN5O2	-40.51	-154.51	108.59	91.21	2.58	19.20	13.64	255.0523	256.0596	254.0450
Acetamiprid	7.06	C10H11ClN4	-41.79	-147.62	110.58	95.12	3.03	17.90	10.37	222.0672	223.0745	221.0599
Thiacloprid	7.72	C10H9ClN4S			120.83		8.97	48.09		252.0236	253.0309	251.0164
Azithromycin	7.99	C38H72N2O12	0.00	-92.18		189.60	5.76	141.42	9.88	748.5085	749.5158	747.5012
Dimethyl phthalate (DMP)	8.11	C10H10O4		0.12	111.47	98.57	3.37	10.05	3.40	194.0579	195.0652	193.0506
Carbamazepine	9.89	C15H12N2O	-43.81	-155.34	112.57	96.02	3.70	17.14	8.65	236.0950	237.1022	235.0877
Erythromycin	10.19	C37H67NO13	-4.99		113.53		4.93	49.91		733.4612	734.4685	732.4540
Dimetamorph (I)	11.52	C21H22ClNO4	-30.63		105.24	93.67	2.57	15.86	13.55	387.1237	388.1310	386.1165
Dimetamorph (II)	11.79	C21H22ClNO4								387.1237	388.1310	386.1165
Diclofenac	12.63	C14H11Cl2NO2	-3.96		119.66		4.63	37.30		295.0167	296.0240	294.0094
Difenoconazole	13.41	C19H17Cl2N3O3	-6.83		103.75	15.06	4.61	36.02	61.09	405.0647	406.0720	404.0574
Diisodecyl phthalate (DiDP)	17.13	C28H46O4	-77.88		67.09		5.07	72.44		446.3396	447.3469	445.3323
Solanine	9.35	C45H73NO15		0.24	193.04	100.54	17.17	31.21	12.02	867.4980	868.5053	866.4907

Tomatine	10.13	C50H83NO21		0.24	110.17	100.54	3.51	23.27	12.02	1033.5458	1034.553	1032.5385
Dopamine	1.42	C8H11NO2		1.62	157.32	86.61	12.45	45.98	31.68	153.0790	154.0863	152.0717
Tyramine	1.99	C8H11NO		0.55	109.13	93.74	2.67	15.77	4.79	137.0841	138.0913	136.0768
Serotonin	2.73	C10H12N2O		0.03	160.27	104.54	3.21	60.93	7.72	176.0950	177.1022	175.0877
Tryptamine	4.67	C10H12N2		-1.12	104.43	99.79	6.53	42.56	7.73	160.1000	161.1073	159.0928
d4-Seratonin	3.99	C10H9D4N2OCl								180.126163		181.1340
d10-Carbamazepine	9.81	C15D10H2N2O								246.173213		247.1689

Data Acquisition Quality and Data processing

No carryover was observed based on multiple injections of solvent and procedure blanks. Data inspection revealed enough quality of target compounds in terms of retention drift (< 10 seconds) and ppm deviation (< 2.5 ppm), confirmed the good analytical performance of MS^e and DDA acquisitions. PCA score plot was also used to check the potential presence of sample outliers in the analysed batch (Figure SI 1) and to verify that blanks and QC samples were grouped together and separately from the study samples.

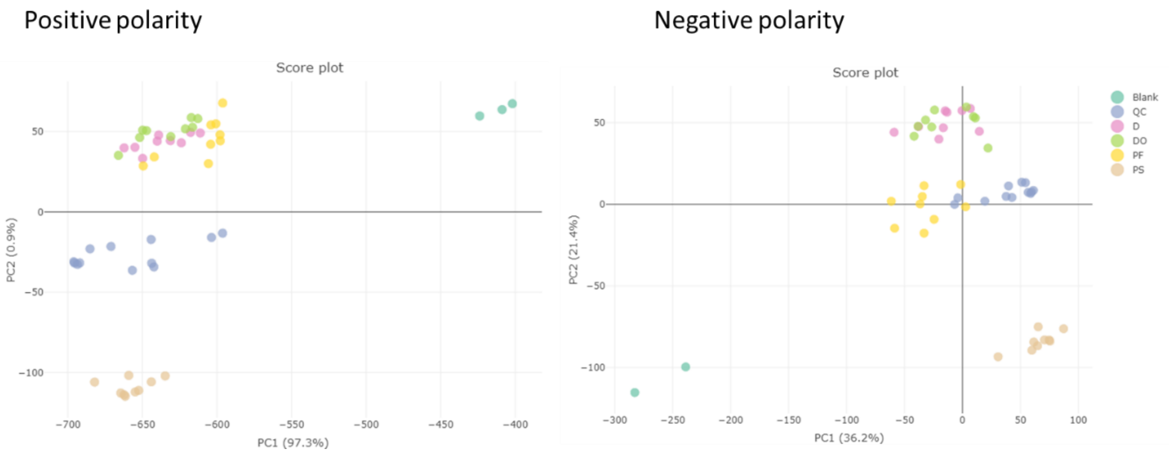

Figure SI 1. The PCA analysis of the whole analyzed sample batch – positive polarity (left) and negative polarity (right). Blank – procedure blank, QC-quality control samples, DO – organically produced "datterini", D – conventionally produced "datterini", PF – plump fresh tomatoes, PS – processed tomatoes.

The optimal parameters for data processing of raw data were selected based on information obtained through target compounds (Table SI 6) using Progenesis QI. All analyses of raw and processed data were performed using R software with in-house written code available on GitHub: [https://github.com/ana-kovacic/NeuroTOm].

Initially, the signal intensity of target compounds showed a correlation with the injection order and a drop in signal within the analyzed batch, which was also confirmed by the drop in internal standard signals during acquisition. To remove analytical drift, different normalization techniques were applied [34], including the probabilistic quotient normalization (PQN) and a linear regression normalization. The PQN normalization method did not remove the analytical drift comparing the relative standard deviation of the signal of mass features in quality controls (QC) samples from raw and PQN normalized data (RSD_{pqn} ~ RSD_{raw}). Finally, the analytical drift was removed using the linear regression normalization on the log transformed intensities, by fitting a linear model to QC samples against the injection order and then applying it to correct the entire dataset (predict values and apply normalization) (Figure SI 2). The missing values were subsequently imputed by replacing them with a random value between zero and one-tenth of the minimum value in the column (minimum area of individual mass features between all samples).

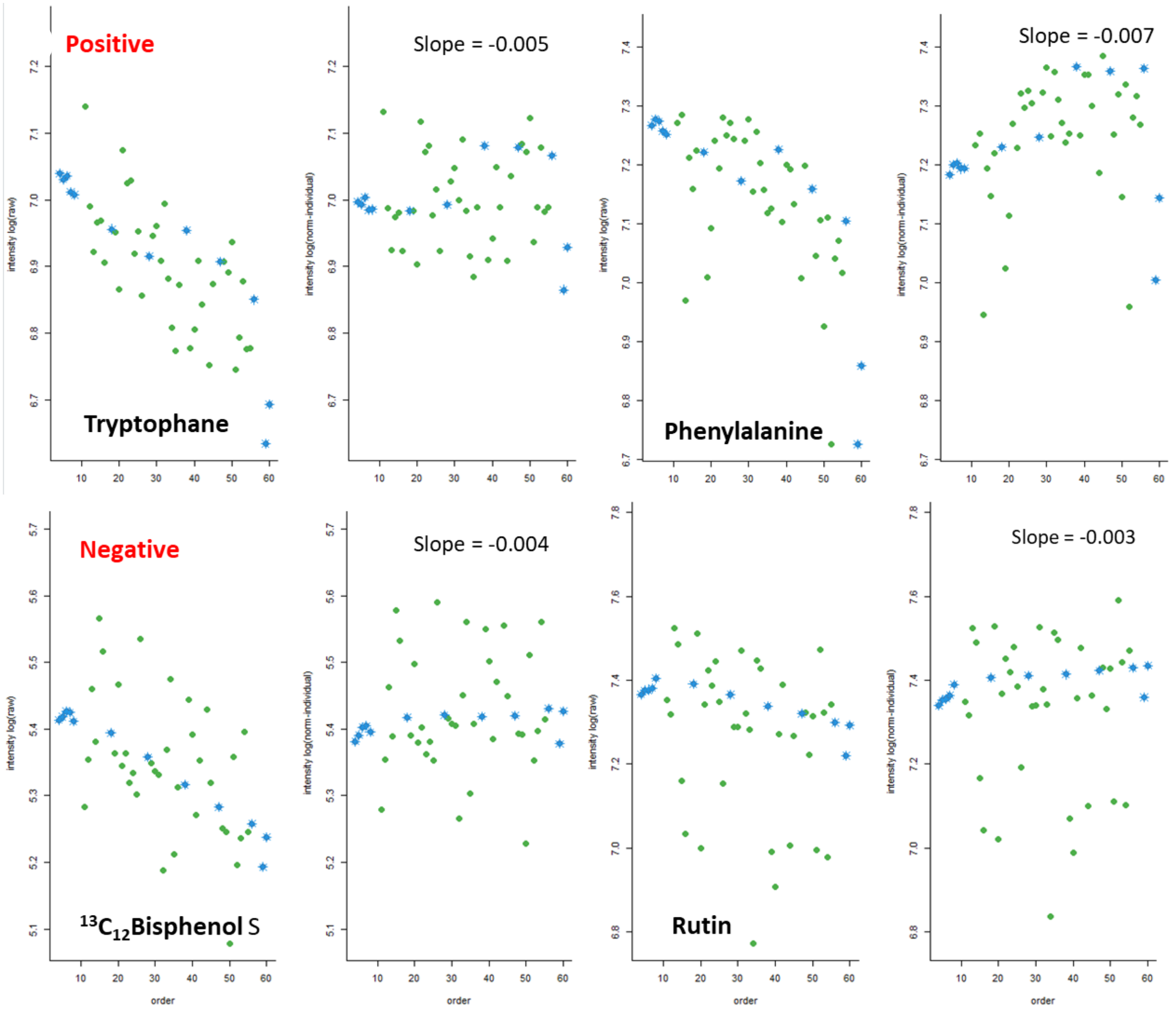

Figure SI 2. Examples of signal intensity of selected target compounds and internal standards in both positive (above) and negative (bellow) modes, extracted from raw data (left), normalized data using linear regression model based on coefficients of individual slope per mass feature (right). The blue and green dots represent the signal intensity (in log10 scale) in QC samples and study samples versus the injection order, respectively. Using the linear normalization model with individual slope (right graphs) was selected because it produced lower RSD values for QC samples compared to raw data during injection.

Following data normalization step, the filtration procedure was applied to remove noisy and irrelevant mass features based on three main conditions. A mass feature was retained in the data set if the feature's intensity in samples was at least 5x higher than in blanks, the signal variability in QC samples was lower than in samples (in terms of relative standard deviation) and if it was present in at least 75% of QC samples. The number of mass features in the original set was reduced from 20,915 to 17,207 in positive polarity and from 14,064 to 9,919 in negative polarity.

Results

Characterization of neuroactive chemical profile in tomatoes

Carotenoids – UPLC-MS-DAD

The determined concentrations of quantified carotenoids (> LOQ) in the tested groups of tomatoes (organic "datterini"; DO, conventional "datterini"; D, plump tomatoes; PF, processed tomatoes; PS) are reported in the Tale SI 1. Each group included six biological replicates (1–6). Additionally, separate

analyses of the tomato peel (Peel) and fruit body (Core) were performed and are also included in the table SI 5.

Table SI 5 The concentration (mg/kg fresh weight) of carotenoids quantified in tested group of tomatoes (organic "datterini"; DO_1-6, conventional "datterini"; D_1-6, plump tomatoes; PF_1-6, process tomatoes; PS_1-6, and tomato peel (Peel_1-3) and fruit pulp (Core_1-3)). The concentration of carotenoids bellow limit of quantification; < LOQ.

Carotenoids	Violaxanthin	Lutein	Neoxanthin	Lycopene	β-Carotene	Phytoene
Sample Name	mg/kg fresh weight					
DO_1	0.12	0.47	0.26	21.68	5.26	34.32
DO_2	0.09	0.34	0.16	20.71	4.05	40.36
DO_3	0.38	1.02	0.73	17.73	6.95	41.46
DO_4	0.28	0.47	0.39	31.07	11.60	58.12
DO_5	0.20	0.59	0.31	27.91	7.07	35.10
DO_6	0.36	0.50	0.93	17.32	5.63	39.74
D_1	0.22	0.51	0.40	26.48	7.77	34.26
D_2	0.30	0.63	0.52	27.66	9.10	51.46
D_3	0.26	0.36	0.56	22.42	4.38	36.70
D_4	0.20	0.45	0.40	43.41	6.92	54.42
D_5	0.26	0.55	0.56	25.82	5.67	33.90
D_6	0.21	0.43	0.48	35.63	5.90	35.75
PF_1	0.27	0.99	0.47	27.30	5.50	67.18
PF_2	0.10	0.44	0.13	31.70	4.77	54.70
PF_3	0.12	0.70	0.62	42.89	27.99	61.11
PF_4	0.11	0.63	0.12	57.20	9.81	18.22
PF_5	0.26	0.97	0.57	21.25	4.31	44.34
PF_6	0.10	0.59	0.29	52.70	2.86	49.65
PS_1	< LOQ	0.23	< LOQ	24.28	4.84	33.61
PS_2	< LOQ	0.21	< LOQ	30.83	3.05	49.79
PS_3	< LOQ	0.55	< LOQ	41.10	9.36	82.72
PS_4	< LOQ	0.43	< LOQ	63.80	1.09	34.84
PS_5	< LOQ	0.27	< LOQ	34.45	5.17	56.64
PS_6	< LOQ	0.47	< LOQ	29.25	4.05	34.29
Peel_1	0.28	0.67	2.07	114.02	47.39	103.48
Peel_2	0.38	0.80	1.53	89.06	31.82	65.78
Peel_3	0.50	1.00	1.26	52.73	20.58	14.33
Core_1	0.09	0.45	0.49	121.38	13.08	61.71
Core_2	0.16	0.63	0.50	80.18	13.58	33.89
Core_3	0.21	0.62	0.37	91.87	16.15	13.80

The results of the pairwise comparison, including the p-value and Cohen's d, were used to compare the presence of quantified carotenoids between organically and conventionally produced tomatoes (DO vs. D), different tomato varieties (D vs. PF), and processing methods (PS vs. PF). Carotenoids were identified as discriminate compound for specific conditions if they met both statistical criteria: a p-value < 0.05 and an effect size > 1 (Table SI 6).

Table SI 6 Statistical analysis – Pairwise comparison (p-value < 0.05, absolute value of Cohen's d > 1) of the quantified carotenoids and tested tomato groups to identify discriminate carotenoids for the difference between organic vs. conventional "datterini" (DO vs. D), "datterini. No data is available in cases where carotenoids were below the LOQ in all samples of the compared group.

Carotenoid	DO vs.D p-value	DO vs.D cd	D vs. PF p-value	D vs. PF cd	PS vs. PF p-value	PS vs. PF cd	discriminate organic	discriminate variety	discriminate process
Violaxanthin	0.9955	0.0034	0.9630	0.0274			FALSE	FALSE	
Lutein	0.0978	1.0708	0.3850	0.5246	0.0014	1.9401	FALSE	FALSE	TRUE
Neoxanthin	0.6772	-0.2522	0.6971	0.2314			FALSE	FALSE	
Lycopene	0.3532	-0.5760	0.9439	0.0422	0.2769	-0.6708	FALSE	FALSE	FALSE

B-Carotene	0.9864	0.0101	0.2531	0.7181	0.6099	0.3042	FALSE	FALSE	FALSE
Phytoene	0.7856	0.1626	0.2918	0.6628	0.4011	-0.5124	FALSE	FALSE	FALSE

Non-target analysis – Neuroactive compounds – LC-QTOF-MS

The experimental MS/MS spectra of the annotated compounds are provided in the attached PDF file: MSMS_NeuroactiveProfileTomatoes. In this file, mirror spectra are shown, with the red fragments corresponding to the ions that matched between the tomato samples and the ions in the MS/MS spectra obtained by the injection of the corresponding chemical standard under the same analytical conditions, whereas blue fragments indicate those ions that matched between the tomato samples and the ions in the MS/MS spectra obtained from used reference libraries. Additionally, experimental MS/MS spectra with no match in the library, annotated at confidence level 3, are provided as individual MS/MS spectra.

The distribution of the annotated compounds across the investigated tomato groups, based on transformed, normalized, and imputed data, is presented in the attached PDF file: DistributionNeuroactiveCompoundsTomatoes. The boxplots in this file represent the normalized intensity values (in log_{10} scale) of each individual compound, as provided in Table SI 7, across all samples within the tested groups (DO, D, PF, and PS).

All data is also available in the MetaboLights repository (accession number: REQ20250205208439, (https://www.ebi.ac.uk/metabolights/editor/study/REQ20250205208439) [17].

Table SI 7 The normalized intensity values (in $\log_{10}$ scale) of each individual annotated compound, across all samples within the tested groups (DO, D, PF, and PS).

**Annotated compounds**	**DO_1**	**DO_2**	**DO_3**	**DO_4**	**DO_5**	**DO_6**	**D_1**	**D_2**	**D_3**	**D_4**	**D_5**	**D_6**	**PF_1**	**PF_2**	**PF_3**	**PF_4**	**PF_5**	**PF_6**	**PS_1**	**PS_2**	**PS_3**	**PS_4**	**PS_5**	**PS_6**
Amino(nitrophenyl)methylsulfonyl-propanoic acid	5.5	5.48	5.29	5.34	5.24	5.31	5.16	5.3	5.22	5.2	5.24	5.38	5.2	5.01	5.04	5.1	5.12	5.22	6.13	5.81	6.11	6.03	5.78	6.25
Aspartame (I)	5.57	5.25	5.29	5.32	5.39	5.43	5.2	5.26	5.32	5.18	5.21	5.25	5.53	5.3	5.58	5.52	5.67	5.53	5.4	5.33	5.32	5.25	5.44	5.25
Aspartame (II)	6.35	6.04	6.33	6.22	6.25	5.91	5.89	6.49	6.15	6.2	6.07	6.19	6.18	5.79	6.42	6.36	6.62	6.02	6.65	6.51	6.56	6.45	6.56	6.52
Hydroquinone	2.72	2.77	2.93	2.2	2.09	2.86	2.59	2.89	2.55	2.14	3.01	2.27	2.9	3.32	3.39	3.05	2.86	3.74	4.95	4.51	4.34	4.7	4.95	4.53
1-Naphthylamine	6.88	7.02	7.41	7.58	7.48	7.18	7.34	7.41	7.53	7.48	7.33	7.59	7.38	7.12	7.25	7.36	7.16	7.61	7.31	7.2	7.29	7.17	7.35	7.22
Derivative of acetaminophen	6.43	6.52	6.4	6.45	6.53	6.7	6.39	6.06	6.32	6.23	6.5	6.56	6.46	6.59	6.19	6.16	6.38	6.44	6.21	5.96	6.1	6.02	6.07	6.19
Difenoconazole	2.29	2.24	1.17	2.15	2.17	2.12	3.25	4.2	3.37	5.1	2.45	6.23	5.18	3.21	1.8	1.93	-0.76	4.88	3.43	3.5	3.25	3.76	0.99	1.99
Methylsulfonyl(pyridin-ylmethyl)piperidinyl-pyridin	4.53	4.47	4.46	4.29	4.25	4.16	5.19	6.43	5.45	4.15	4.32	7.15	7.53	5.27	5.79	4.7	4.57	4.33	5.4	5.18	4.83	5.13	4.61	5.08
Glutamine	5.18	4.63	5.19	5.01	5.35	5.27	4.7	5.21	5.07	5.17	5.45	5.06	5.23	5.73	5.29	5.7	5.28	5.54	3.56	3.19	4.12	3.47	3.55	3.71
Glutamylphenylalanine	5.47	5.49	5.45	5.49	5.54	5.55	5.55	5.79	5.34	5.6	5.86	5.48	5.94	4.93	5.81	5.31	5.43	5.76	6.02	5.8	5.92	5.82	5.86	5.99
Guanine	7.6	7.56	7.63	7.63	7.67	7.62	7.49	7.61	7.63	7.59	7.6	7.66	7.55	7.48	7.51	7.5	7.55	7.62	7.52	7.49	7.53	7.45	7.61	7.56
Leucine-leucine (I)	4.59	4.55	4.65	4.85	4.82	4.75	5.1	5.22	4.81	5.14	5.38	4.71	5.61	4.45	5.48	5.01	4.78	5.36	6.06	5.88	6.15	5.98	5.72	6
Leucine-leucine (II)	4.03	4.14	4.18	4.28	4.27	3.95	4.49	4.78	4.34	4.79	4.57	4.31	5.3	4.02	5.49	4.85	4.95	4.77	5.71	5.38	5.67	5.5	5.43	5.71
Leucylvaline	5.67	5.77	5.79	5.81	5.94	5.9	6.02	6.17	5.79	5.98	6.21	5.82	6.31	5.25	6.32	5.72	5.91	6.13	6.75	6.49	6.66	6.53	6.48	6.6
N-acetyltryptophan	6.27	6.27	6.15	6.27	6.07	6.4	6.33	6.21	6.2	6.04	6.51	6.2	6.62	6.47	5.8	6.24	6.02	6.88	6.67	6.66	6.58	6.75	6.67	6.63
Phenylalanine	7.23	7.02	7.27	7.3	7.32	7.14	6.95	7.32	7.3	7.24	6.96	7.27	7.15	7.11	7.3	7.27	7.35	7.19	7.25	7.32	7.36	7.31	7.38	7.34
Tryptophan	7.13	6.98	7.12	6.99	7.07	7.12	6.92	7.08	6.98	6.88	6.99	6.99	6.98	6.9	6.92	6.92	6.94	6.91	6.99	7.02	7.09	6.98	7.04	6.94
Tyramine	5.46	5.71	5.63	5.67	5.71	6	5.77	5.46	5.61	5.57	5.82	5.82	5.41	5.24	5.11	5.25	5.14	5.59	5.35	5.42	5.42	5.42	5.56	5.48
10,16-Dihydroxypalmitic acid	4.97	3.34	4.45	4.49	4.9	4.58	4.79	4.74	5.04	4.76	4.6	4.35	4.27	4.86	4.57	3.73	4.61	4.64	6.06	5.96	5.17	5.9	5.46	5.8
2-Isopropylmalic acid	7.03	6.66	6.85	6.79	6.68	6.98	6.47	7.14	6.68	6.76	6.62	6.75	6.54	6.3	6.87	6.95	7	6.96	6.81	6.55	6.49	6.6	6.89	6.39
Elaidic/Oleic acid	5.66	5.52	5.33	5.34	5.21	5.21	5.38	5.34	5.31	5.32	5.28	5.35	5.41	5.34	5.43	5.34	5.99	5.3	5.98	6.07	6.06	5.89	6.16	5.76
Hydroxyadipic acid	6.26	6.27	6.64	6.45	6.34	6.03	6.21	6.77	6.21	6.38	5.84	6.04	5.93	5.83	5.87	6.17	5.99	6.21	5.85	5.59	5.39	5.65	5.88	5.56
Linoleic acid	4.63	4.09	3.61	3.23	2.83	2.4	3.92	3.98	3.34	3.36	2.48	3.4	4.27	3.52	4.85	3.32	5.74	3.26	6.73	6.43	6.26	5.93	5.97	5.29
Adenosine	7.77	7.81	7.66	7.75	7.76	7.77	7.63	7.77	7.7	7.72	7.79	7.79	7.88	7.76	7.83	7.82	7.79	7.76	7.89	7.86	7.87	7.87	7.94	7.94
Dopa	5.46	5.45	5.48	5.61	5.59	5.62	5.32	5.48	5.51	5.5	5.53	5.64	5.47	5.51	5.63	5.54	5.9	5.76	5.48	5.59	5.54	5.56	5.56	5.73
Glutamic acid	6.74	6.67	7	6.95	6.95	6.77	6.53	6.98	6.88	6.93	6.88	7	6.67	6.64	6.69	6.62	6.81	6.83	6.82	6.77	6.92	6.81	6.75	6.94
Serotonin	6.37	6.55	6.22	6.39	6.31	6.67	6.53	6.28	6.29	6.33	6.58	6.4	6.34	6.05	6.25	6.2	6.32	6.11	5.94	5.71	6.2	5.82	6.01	5.88

Tryptamine	5.69	6.29	6.53	6.57	6.49	6.39	6.4	6.51	6.58	6.47	6.57	6.6	6.32	5.99	6.32	6.19	6.15	6.63	6.08	6.12	6.2	6.09	6.23	6.04
Tyrosine	7.28	6.77	7.08	6.91	7.03	7.15	6.62	7.12	7.05	6.94	6.3	7.04	6.8	6.99	6.95	7.06	6.95	7.21	7.06	7.08	7.08	7.11	7.12	7.12
Adenine	6.76	7.03	6.92	7.00	6.8	6.93	6.96	7.00	6.87	7.07	6.97	7.03	6.84	6.48	6.42	6.45	6.28	6.9	6.93	6.74	6.81	6.92	6.92	6.91
Adenosine monophosphate	6.96	7.12	7.09	7.11	6.97	7.02	7.09	7.19	7.1	7.22	7.02	7.13	7.04	6.8	6.89	6.87	6.58	7.06	7.02	7.00	7.06	7.07	7.04	7.07
Guanosine 5'-monophosphate (I)	5.15	5.53	5.49	5.53	5.28	5.49	5.66	5.65	5.48	5.88	5.7	5.64	5.56	5.16	5.2	5.16	4.64	5.66	5.37	5.29	5.43	5.57	5.36	5.55
Guanosine 5'-monophosphate (II)	4.9	5.36	5.58	5.53	5.19	5.59	5.99	5.52	5.3	5.62	5.48	5.78	5.33	4.47	4.33	4.36	3.66	5.13	5.53	5.51	5.54	5.76	5.56	5.7
Uridine 5'-monophosphate	6.7	6.63	6.84	6.73	6.65	6.79	6.68	7.11	6.71	7.12	6.74	6.9	6.8	6.49	6.77	6.72	6.28	7.04	7.09	6.97	7.03	7.11	7.21	7.08
5-caffeoylshikimic acid	4.44	4.59	4.57	4.99	4.80	4.61	4.41	4.71	4.42	4.58	5.11	4.77	5.18	4.75	4.72	5.20	4.38	4.81	4.70	4.50	4.52	4.82	4.59	4.77
Ascorbic Acid	6.91	6.81	6.75	6.89	6.78	6.84	6.78	6.79	6.83	6.77	6.62	6.88	6.64	6.66	6.73	6.67	6.85	6.56	6.65	6.42	6.72	6.5	6.66	6.36
Caffeic acid	5.51	5.59	5.5	5.56	5.59	5.55	5.59	5.65	5.86	5.63	5.68	5.55	5.84	5.96	5.97	6.04	5.65	6.30	6.26	6.16	6.07	6.21	6.26	6.14
Chlorogenic Acid	6.75	7.08	6.9	7.08	6.88	7.19	7.21	6.87	6.79	6.78	7.22	7.03	6.89	6.66	6.21	6.74	6.31	6.48	6.74	6.75	6.61	6.80	6.70	6.78
Citric acid	7.47	7.69	7.56	7.71	7.5	7.55	7.76	7.54	7.6	7.6	7.75	7.62	7.72	7.48	7.36	7.25	7.40	7.51	7.65	7.64	7.64	7.74	7.68	7.64
Crepenynic acid	4.24	4.04	2.89	2.53	2.38	1.13	2.95	3.27	0.43	2.86	0.59	2.85	4.11	2.14	4.34	2.23	5.30	2.75	6.14	5.85	5.64	5.21	5.23	4.49
Cryptochlorogenic acid	6.67	7.06	6.98	7.12	7.02	7.09	7.15	6.58	6.96	6.85	7.16	7.02	6.87	6.75	6.18	6.38	6.26	6.63	6.48	6.61	6.57	6.59	6.50	6.59
Dihydrocoumarin	6.18	5.94	6.17	6.25	6.28	6.06	5.9	6.26	6.21	6.2	5.9	6.21	6.12	6.07	6.27	6.27	6.34	6.15	6.24	6.26	6.32	6.25	6.36	6.32
Eriodictyol (I)	4.72	4.68	4.13	4.47	4.7	4.51	5.21	4.07	4.78	4.3	5.83	4.66	4.76	5.15	5.51	4.57	4.57	5.52	5.79	5.79	5.64	5.82	5.63	5.3
Eriodictyol (II)	5.18	5.49	4.67	5.82	5.42	5.22	5.92	4.73	5.96	5.43	6.31	4.98	5.77	5.29	5.61	4.09	5.14	5.02	2.4	-0.97	1.75	2.27	2.66	1.98
Eriodictyol (III)	5.52	5.51	4.71	4.51	4.53	4.91	4.46	4.11	4.84	4.33	5.09	4.95	2.85	4.01	1.91	3.61	3.06	3.56	5.66	5.9	5.64	5.65	5.90	5.30
Ferulic acid	5.9.0	6.00	5.65	5.74	5.71	6.01	6.22	5.81	5.84	5.72	6.26	5.69	6.37	6.17	6.11	6.5	6.11	6.13	5.66	5.73	5.36	5.98	5.47	5.92
Feruloyltyramine	5.24	4.88	5.13	4.63	5.10	5.49	5.11	5.44	5.09	4.98	4.32	4.09	5.67	3.71	4.17	5.13	4.39	4.91	5.99	5.81	5.67	5.74	5.74	5.36
Hesperetin	4.30	4.45	3.79	4.47	5.10	4.16	5.81	2.91	4.18	4.24	6.19	4.79	5.31	6.01	5.21	2.99	5.61	4.86	5.59	5.89	5.98	5.9	5.67	5.36
Homoeriodictyol	4.74	5.62	4.32	5.07	5.20	4.53	5.3	3.87	4.67	4.01	6.61	4.59	5.13	5.3	3.94	2.02	4.23	3.48	2.85	2.56	3.03	2.84	2.43	2.56
Hydroxycinnamic acid	5.54	4.70	5.20	4.74	4.85	5.00	4.64	5.26	5.18	5.07	4.07	4.95	5.04	5.63	5.34	5.71	5.2	5.9	5.05	5.02	4.95	5.02	5.05	5.10
Isoquercetin	6.89	7.18	6.94	7.05	6.9	6.99	7.09	7.00	7.06	7.06	7.26	7.07	6.57	6.37	6.52	6.18	6.4	6.53	6.77	6.85	6.83	6.77	6.83	6.54
Malonyltryptophan	5.54	5.77	5.75	5.73	5.44	5.79	5.37	5.79	5.69	5.18	5.86	5.72	5.81	5.42	4.66	5.01	4.98	6.15	5.88	5.89	5.74	5.93	5.88	5.78
Naringenin	7.44	7.61	7.05	7.65	7.51	7.51	7.7	7.09	7.72	7.32	8.06	7.26	7.76	7.55	7.62	6.74	7.15	7.32	4.96	4.94	4.87	4.9	4.39	4.29
Naringenin chalcone	6.45	6.52	5.82	6.48	6.45	6.17	6.71	5.99	6.63	6.2	7.26	6.11	6.9	6.76	6.76	5.67	5.75	6.51	7.3	7.28	7.24	7.31	7.02	6.83
Naringin	4.11	2.96	3.06	3.41	3.09	3.53	3.5	3.21	2.95	3.43	2.28	2.96	3.13	3.17	3.13	3.38	3.89	3.67	3.99	3.86	3.93	3.65	4.04	3.66
Neochlorogenic Acid	6.23	6.16	6.16	6.14	6.03	6.15	5.91	6.14	6.2	6.22	5.69	6.14	5.41	5.27	5.34	5.32	5.69	5.77	6.77	6.79	6.50	6.75	6.86	6.71

Phenyllactic acid	2.84	2.79	2.65	2.84	2.52	2.67	2.78	2.18	2.79	1.96	2.79	2.2	3.08	2.15	3.4	3.3	3.21	2.26	6.28	6.12	6.15	6.22	6.19	5.52
Quercetin	4.53	4.14	3.18	3.73	4.23	4.3	4.38	3.26	4.83	3.58	4.73	3.59	3.54	3.79	3.3	2.48	3.53	3.32	5.44	5.25	4.76	5.22	4.73	4.15
Quinic Acid	7.15	7.23	7.09	7.2	7.1	7.28	7.24	7.18	7.07	7.25	7.29	7.11	7.03	6.87	6.87	6.87	6.92	7.21	7.05	6.97	6.92	7.03	7.17	7.00
Resveratrol	2.22	2.28	2.39	2.98	2.24	2.4	3.32	2.76	3.59	3.12	3.25	-2.09	3.44	3.8	3.54	3.55	3.74	3.41	1.99	-1.14	2.36	-0.8	2.72	-0.78
Rutin	7.35	7.53	7.37	7.48	7.33	7.43	7.52	7.42	7.48	7.51	7.59	7.47	7.17	7.02	7.19	6.84	6.99	7.1	7.32	7.38	7.38	7.34	7.36	7.11
5'-Deoxy-5'-(methylthio)adenosine	6.37	6.1	6.28	6.4	6.4	6.24	6.15	6.00	6.26	6.01	6.21	6.14	6.53	5.99	6.4	6.36	6.43	6.39	7.68	7.72	7.75	7.74	7.64	7.82
N-Caffeoyl putrescin	6.48	6.51	6.7	6.57	6.72	6.23	6.37	6.97	6.86	6.55	7.02	6.44	6.91	6.48	6	6.21	5.63	5.93	7.09	6.89	6.82	6.79	6.73	6.97
N-Feruloylputrescine	6.54	7.03	6.7	6.8	6.86	6.34	6.52	7.25	6.69	6.87	7.36	6.68	7.31	6.32	6.53	6.71	6.47	6.82	7.13	7.06	6.99	6.97	6.88	6.96
Sibricose A3	4.97	4.87	4.57	4.57	4.42	4.62	4.38	4.62	4.71	4.56	4.75	4.7	5.61	5.26	5.01	4.8	4.68	5.64	5.31	5.56	5.46	5.33	5.38	5.27
Tomatidinol (I)	5.89	5.75	5.85	5.64	5.53	6.04	5.97	5.38	5.61	5.61	5.47	5.86	5.53	5.07	5.44	5.51	5.7	5.42	6.5	6.68	6.5	6.58	6.29	6.49
Tomatidinol (II)	5.23	5.14	5.14	4.92	4.64	5.44	5.31	4.69	4.87	4.83	4.57	5.14	4.74	4.28	4.68	4.71	4.78	4.66	6.04	6.22	6.04	6.11	5.85	6.01
Tomatine	6.52	6.92	6.72	6.78	6.35	6.81	6.77	6.56	6.65	6.46	6.96	6.77	7.07	5.68	6.2	6.54	5.92	6.21	7.31	7.47	7.07	7.36	6.97	6.96
4-Pyridoxic acid	6.12	5.91	5.92	6.00	5.89	5.75	5.62	5.95	6.00	5.73	5.6	5.97	5.19	5.58	6.05	6.05	6.05	6.00	6.12	6.13	6.1	6.16	6.21	5.94
Pantothenic Acid	6.94	6.92	6.9	6.95	6.96	7.08	6.79	6.88	6.88	6.73	6.93	6.95	6.91	6.67	6.81	6.67	6.95	7.00	6.72	6.74	6.74	6.71	6.78	6.72

The trends in the data were explored using PCAs on the whole normalized and filtered mass feature dataset (Figure SI 3). The results suggest a bigger difference between differently processed tomatoes followed by a difference between different type of tomatoes, rather than differently produced tomatoes.

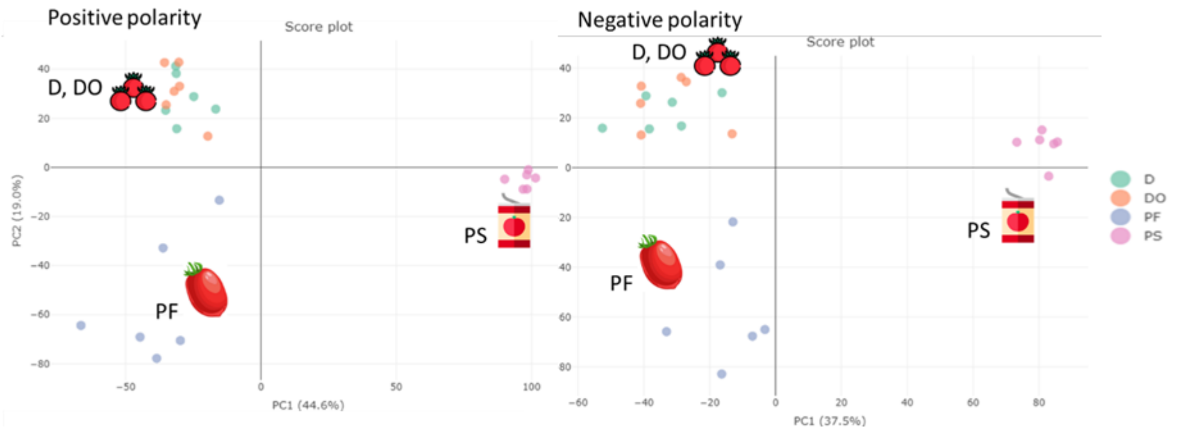

Figure SI 3. PCA analysis of study sample data (organic "datterini" – DO, conventional "datterini" – D, fresh plump tomato – PF, processed tomato – PS) performed on the feature list obtained after normalization and filtering processes for data acquired in positive (right) and negative (left) polarity mode.

References

17 Yurekten O, Payne T, Tejera N, et al. MetaboLights: open data repository for metabolomics. *Nucleic Acids Res*. 2024;52(D1):D640-D646. doi:10.1093/nar/gkad1045

34 Winter B. Linear Models and Linear Mixed Effects Models in R with Linguistic Applications. https://doi.org/10.48550/arXiv.1308.5499
